# Dynamics of Segregation and Integration in Directional Brain Networks: Illustration in Soldiers With PTSD and Neurotrauma

**DOI:** 10.3389/fnins.2019.00803

**Published:** 2019-08-23

**Authors:** D. Rangaprakash, Michael N. Dretsch, Jeffrey S. Katz, Thomas S. Denney Jr., Gopikrishna Deshpande

**Affiliations:** ^1^Department of Electrical and Computer Engineering, AU MRI Research Center, Auburn University, Auburn, AL, United States; ^2^Departments of Radiology and Biomedical Engineering, Northwestern University, Chicago, IL, United States; ^3^U.S. Army Aeromedical Research Laboratory, Fort Rucker, AL, United States; ^4^U.S. Army Medical Research Directorate-West, Walter Reed Army Institute for Research, Joint Base Lewis–McChord, WA, United States; ^5^Department of Psychology, Auburn University, Auburn, AL, United States; ^6^Alabama Advanced Imaging Consortium, Auburn, AL, United States; ^7^Center for Neuroscience, Auburn University, Auburn, AL, United States; ^8^Center for Health Ecology and Equity Research, Auburn University, Auburn, AL, United States; ^9^Department of Psychiatry, National Institute of Mental Health and Neurosciences, Bengaluru, India

**Keywords:** functional MRI, network dynamics, complex network modeling, effective connectivity, dynamic connectivity, posttraumatic stress disorder, mild traumatic brain injury, machine learning

## Abstract

Brain functioning relies on various segregated/specialized neural regions functioning as an integrated-interconnected network (i.e., metastability). Various psychiatric and neurologic disorders are associated with aberrant functioning of these brain networks. In this study, we present a novel framework integrating the strength and temporal variability of metastability in brain networks. We demonstrate that this approach provides novel mechanistic insights which enables better imaging-based predictions. Using whole-brain resting-state fMRI and a graph-theoretic framework, we integrated strength and temporal-variability of complex-network properties derived from effective connectivity networks, obtained from 87 U.S. Army soldiers consisting of healthy combat controls (*n* = 28), posttraumatic stress disorder (PTSD; *n* = 17), and PTSD with comorbid mild-traumatic brain injury (mTBI; *n* = 42). We identified prefrontal dysregulation of key subcortical and visual regions in PTSD/mTBI, with all network properties exhibiting lower variability over time, indicative of poorer flexibility. Larger impairment in the prefrontal-subcortical pathway but not prefrontal-visual pathway differentiated comorbid PTSD/mTBI from the PTSD group. Network properties of the prefrontal-subcortical pathway also had significant association (*R*^2^ = 0.56) with symptom severity and neurocognitive performance; and were also found to possess high predictive ability (81.4% accuracy in classifying the disorders, explaining 66–72% variance in symptoms), identified through machine learning. Our framework explained 13% more variance in behaviors compared to the conventional framework. These novel insights and better predictions were made possible by our novel framework using static and time-varying network properties in our three-group scenario, advancing the mechanistic understanding of PTSD and comorbid mTBI. Our contribution has wide-ranging applications for network-level characterization of healthy brains as well as mental disorders.

## Introduction

The dynamic abilities of the human brain are attributed to its highly interconnected neural architecture. Functional MRI (fMRI) connectivity modeling is popularly employed to study interrelationships between brain regions at the systems-level. However, fMRI connectivity is limited in that it can characterize only pairwise relationships (i.e., bivariate). To characterize connection ensembles (Rubinov and Sporns, 2010), and not just connection pairs, strategies beyond traditional connectivity modeling, such as complex-network modeling (Rubinov and Sporns, 2010) using graph-theoretic techniques, are useful. This approach makes use of individual connectivity weights as well as the pattern in which these connections coexist, to make various inferences on the network structure.

### Functional Segregation

A graph comprises of a set of nodes (brain regions) that are interconnected by edges (connectivity weights), and network measures quantify different characteristics of the topology of such graphs. Rubinov and Sporns (2010) illustrate the applicability and interpretation of several complex-network measures in brain imaging. Among them, *functional segregation*, necessary for optimal specialized processing, informs about dense-connectedness within separate subnetworks. It quantifies whether the regions connected to a given node are connected amongst themselves, thus forming subnetworks wherein majority of the nodes are connected to every other node. For example, during altered consciousness, segregation is reduced, especially in the thalamus (Crone et al., 2013). In the current study, we employed transitivity (global whole-brain-level measure), clustering coefficient and local efficiency (both local node-level measures) to quantify segregation (Rubinov and Sporns, 2010).

### Functional Integration

In contrast, functional integration captures the ease of interaction between segregated regions (Rubinov and Sporns, 2010). For example, there is elevated segregation in prefrontal and cerebellar subnetworks in attention-deficit hyperactivity disorder (ADHD), but lower integration between these subnetworks (Lin et al., 2014), indicative of characteristic attentional reaction-time deficits observed in this population. In the current study, we employed global efficiency (global measure), shortest path length, and edge betweenness (both local measures at connection-level) to quantify integration (Rubinov and Sporns, 2010). Although traditional connectivity identifies standalone aberrant connections in clinical groups, these measures of integration identify those connections that are not only important by themselves, but are also important for the rest of the connections in the network.

### Graph Measures and Military Population

It has been extensively demonstrated that segregation and integration are disrupted in psychiatric disorders [for example, see (Yu et al., 2013; Rocca et al., 2014)]. Most report a narrow, but balanced relationship between them in healthy populations (called metastability) (Hellyer et al., 2015), which is impaired in neurologic and psychiatric disorders (Yu et al., 2013; Rocca et al., 2014). Using resting-state fMRI and our novel framework, we investigated network-level aberrations in soldiers with posttraumatic stress disorder (PTSD) and post-concussion syndrome (PCS) associated with documented mild traumatic brain injury(s) (mTBI). PCS is an outcome of mTBI, in which the individual presents persistent post-concussive symptoms 3 months’ post-injury.

In military populations, there is considerable comorbidity between mTBI and PTSD (Hoge et al., 2008, 2009), often attributed to life-threatening events such as exposure to blast from improvised explosive devices (IEDs), which result in mTBI as well as psychological trauma. With the prevailing clinical approaches focusing on patient reporting, and with substantial overlapping symptoms between PTSD and PCS (Eierud et al., 2014), a better comprehension of the neurobiological-mechanistic basis for PTSD and PCS is imperative for improved diagnosis and treatment outcomes, and for making return-to-duty decisions. Prior fMRI works on comorbid PTSD and mTBI are limited (Spielberg et al., 2015), although its prevalence is considerably high in general society as well as military populations (Veterans, 2015). In the current study, we explored our novel framework involving functional segregation and integration in three groups: soldiers with elevated posttraumatic stress symptoms (PTSD group), PCS + PTSD (comorbid group sustaining both PTSD and PCS), and healthy combat controls. For the sake of disambiguation, we call complex-network modeling as “network-level,” while connectivity modeling is termed “connectivity-level” and activation analysis as “region-level.” Although several studies have identified region-level and connectivity-level aberrations in specific key prefrontal and subcortical regions in mTBI and PTSD (Simmons and Matthews, 2012), a thorough understanding of the aberrations of directional relationships and associated changes in network structure have not emerged from them. We address this limitation in this study.

### Effective Connectivity

Graph-theoretic analysis begins from network graphs constructed using pairwise connections, which can be obtained through connectivity modeling. Although functional connectivity (FC) has been the predominant choice so far, we sought to investigate directional networks with causal relationships instead of co-activation (a non-directional entity). It has not been adequately explored, even though it is an equally important mechanism for network-level interactions. Causal connectivity has been discovered even in fMRI timescales (Roebroeck et al., 2005; Abler et al., 2006; David et al., 2008; Deshpande et al., 2011; Deshpande and Hu, 2012; Ryali et al., 2016; Rangaprakash et al., 2018a), indicating that identifying causal networks in addition to co-activation networks is important for a more extensive characterization. Further, PTSD and PCS are typically considered as prefrontal dysregulation disorders (Simmons and Matthews, 2012), meaning that prefrontal causal connectivity is compromised. This provided the impetus for us to further investigate directional connectivity. To our surprise, there have been hardly any fMRI studies investigating effective connectivity (EC) in either PTSD or PCS or the comorbid condition.

### Granger Causality

Granger causality (GC), an exploratory technique, was employed to quantify EC (Deshpande et al., 2010b). It is the most prevalent technique for deriving causal relationships in natural systems (Kirchgässner et al., 2012). Both recent simulations (Ryali et al., 2011; Wen et al., 2013) and experimental results, including optogenetics and electrophysiology (David et al., 2008; Katwal et al., 2013; Ryali et al., 2016; Wang et al., 2016), demonstrate that GC is reliable for drawing inferences regarding directional relationships between brain regions when used after deconvolving the hemodynamic response function (HRF) from fMRI data (as done in the current study). Several recent fMRI works have also employed this technique (Deshpande et al., 2013; Sathian et al., 2013; Grant et al., 2014; Lacey et al., 2014; Wheelock et al., 2014; Feng et al., 2015; Grant et al., 2015; Hutcheson et al., 2015; Bellucci et al., 2016).

### Dynamic Connectivity

Most studies investigating fMRI connectivity assume connectivity as stationary over time, although static connectivity does not capture dynamic variations of connectivity. While an fMRI scan endures for several minutes, mental processes occur within a few milliseconds to a few seconds’ time, implying that connectivity varies over the timescales of fMRI scans, and that those variations contain biologically relevant information (Hutchison et al., 2013), which are different from that contained in static connectivity (Jia et al., 2014). Recent works have found connectivity dynamics to be a unique and important marker of brain functioning (Hansen et al., 2015; Jin et al., 2017). Therefore, the current study utilized both static EC (SEC) and dynamic EC (DEC). Brain networks were constructed from strength (SEC) and temporal variability (DEC) of directional connectivity, using which we obtained strength and variability of segregation/integration measures, respectively. Such a characterization of dynamic network properties is one of the important novel contributions of this work. While dynamic connectivity has prevailed in neuroimaging for some time (Hutchison et al., 2013), for the first time we introduce dynamic modeling of segregation and integration in a novel framework.

Lower variability of connectivity over time is associated with both psychiatric and neurologic conditions (Garrett et al., 2013; Jia et al., 2014; Miller et al., 2016; Rashid et al., 2016; Rangaprakash et al., 2017a, 2018a), often corresponding to a lack of cognitive flexibility. Compromised behavioral performance is linked with reduced temporal variance of connectivity in both clinical and non-clinical populations (Sakoğlu et al., 2010; Jia et al., 2014; Rangaprakash et al., 2017a, 2018a). Such reduction is linked to impaired ability in dynamically adjusting to changing conditions (thoughts, behaviors, etc.). A healthy biological system is flexible in response to continual momentary changes within the internal and external milieu of the organism. In those terms, temporally “frozen” connectivity and/or complex-network properties point to compromised brain health. Such a characterization has been done in recent connectivity studies (Jia et al., 2014; Rangaprakash et al., 2017a). Higher variability of connectivity is also considered a marker of greater mental flexibility (Zhang et al., 2016).

### Hypotheses

**FIGURE 1:**
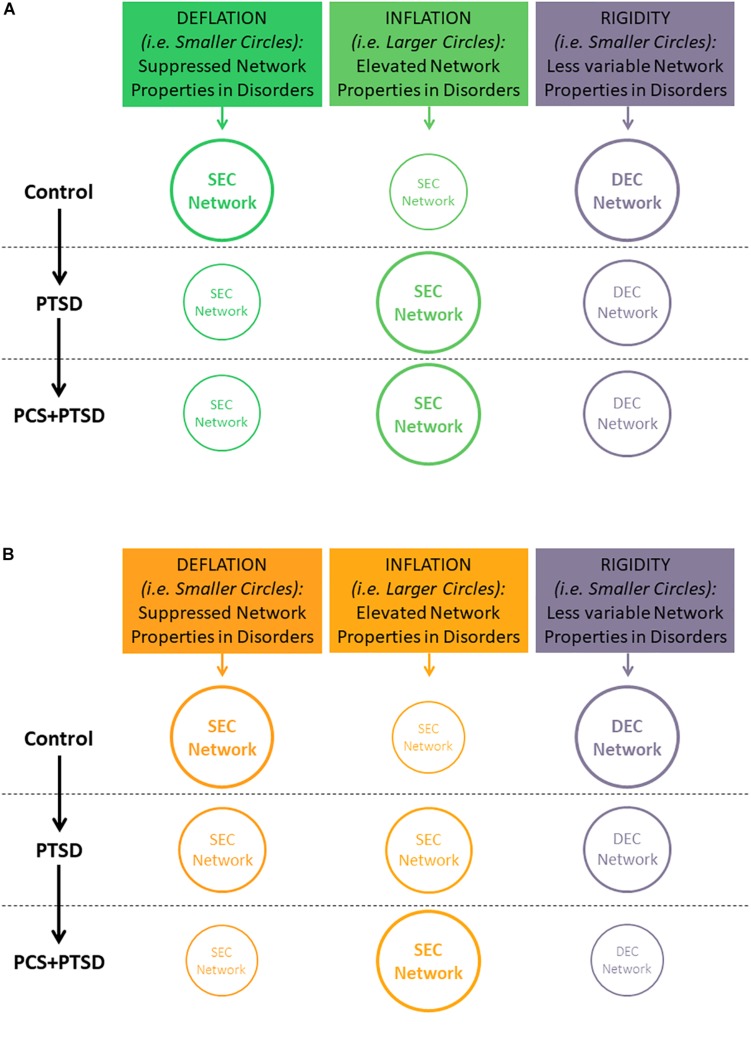
Illustration of our hypothesis showing decreasing temporal variability (implying rigidity) of segregation/integration, and either increasing or decreasing segregation/integration (implying *inflation* or *deflation*, respectively) as we move from Control to PTSD to PCS + PTSD. Font and circle sizes are symbolic of the increasing/decreasing trend, with smaller circles/text representing *deflation* and rigidity, and larger circles/text representing *inflation* and flexibility. **(A)** Hypothesis-1: some network properties would be disrupted only in PTSD (significant for Control vs. PTSD and Control vs PCS + PTSD comparisons, but not PTSD vs. PCS + PTSD comparison). **(B)** Hypothesis-2: some network properties would be significantly different between all three groups. Note that *inflation* and *deflation* generally correspond to elevation or suppression of network properties, respectively, and not just connection strengths of individual paths. However, in the special case when local network properties of the paths are considered, *inflation* and *deflation* are referred to the network properties, as well as connection strengths of the paths under consideration.

In this work, we extend these concepts to the reduced temporal variability (or rigidity) in network properties instead of individual connection strengths. We hypothesized that *PTSD and mTBI are characterized by altered strength and lower temporal variability of segregation and integration in directional brain networks*. We associated the connections exhibiting suppressed network properties with *deflation*, given that reduced engagement of certain prefrontal-subcortical and prefrontal-cortical pairwise connectivities may be considered as an outcome of impaired regulation from prefrontal regions (Gross, 2014). Similarly, we associated the connections exhibiting elevated network properties with *inflation*, or pathologically enhanced network-level engagement, given that pairwise hyper-connectivity is seen as an outcome of neurological disruption (Hillary et al., 2015), and has been noticed in PTSD (Hayes et al., 2012; Simmons and Matthews, 2012; Cisler et al., 2014). Within this framework, we sought to identify such networks properties which were (i) affected by PTSD but not mTBI (we call this *hypothesis-1*, see Figure 1A), and (ii) affected by PTSD as well as comorbid PTSD and mTBI (we call this *hypothesis-2*; see Figure 1B). Such dichotomy would enable us to identify both common (*hypothesis-1*) and distinguishing (*hypothesis-2*) network features between PTSD and mTBI, given the high comorbidity and overlapping symptomatology between them (Spielberg et al., 2015). Notably, we tested the hypothesis on whole-brain data, in a data-driven manner without imposition of any priors, using resting-state fMRI, which is not task dependent. With the network properties that fit our hypothesis, we assessed their association with relevant behaviors (neurocognitive functioning, and symptom severity in PTSD and PCS).

### Machine Learning

Statistical group separation is the analysis framework for our hypothesis. However, statistical separation does not automatically attribute them with predictive diagnostic ability (Deshpande et al., 2010a) at the individual-subject level. Machine-learning classifiers have been successfully utilized on fMRI data for such diagnostic prediction in disorders like major depression (Deshpande et al., 2009), PTSD (Liu et al., 2015), Parkinson’s (Marquand et al., 2013), dementia (Chen et al., 2011), ADHD (Deshpande et al., 2015), prenatal-cocaine-exposure syndrome (Deshpande et al., 2010a), autism (Deshpande et al., 2013; Libero et al., 2015), and many others. However, to the best of our knowledge, there have been no works utilizing complex-network properties in PTSD/mTBI classification. Given the unique high-level information contained in network properties, we expected network measures to possess predictive ability. Neuropsychiatric conditions such as PCS and PTSD are currently diagnosed through clinical observation and self-report, hence classification using neuroimaging-based network signatures can be useful in obtaining more accurate diagnoses in these highly comorbid conditions. Hence, we employed a machine learning technique to identify highly predictive features by recursively eliminating unimportant complex-network features in a data-driven way. In addition, we sought to find an overlap between connections with network properties satisfying our primary hypotheses (Figure 1), *and* those possessing high predictive ability. As our secondary hypothesis, we hypothesized that such network properties would predict the diagnostic membership of a new subject better than available non-imaging measures (neurocognitive, behavioral and self-report measures), thus underscoring their relevance to the underlying neuropathology of mTBI and PTSD. We place special emphasis on network properties having all the desirable qualities assessed in this work: high statistical separation, behavioral relevance and high predictive ability. Our study illustrates the utility of our methodological framework using the PTSD/mTBI cohort as an example.

## Materials and Methods

A schematic of the entire processing pipeline is available at the end of the methods section (Figure 4).

### Participants

Active-duty soldiers (aged between 18 and 50 years) were recruited from Fort Benning, GA, United States and Fort Rucker, AL, United States to participate voluntarily in the study. The study was conducted in accordance with the Declaration of Helsinki, and the procedures were approved by Auburn University’s Institutional Review Board (IRB) as well as the Headquarters United States Army Medical Research and Materiel Command, IRB (HQ USAMRDC IRB). Written informed consent was obtained from all participants.

Eighty-seven male, active duty U.S. Army soldiers were enrolled in the study, which included 17 with PTSD, 42 with comorbid PCS and PTSD (PCS + PTSD), and 28 combat controls (all groups were matched in age, education and race), all having combat experience in Iraq (Operation Iraqi Freedom, OIF) and/or Afghanistan (Operation Enduring Freedom, OEF). Participants were grouped based on symptom severity in PTSD using the “PTSD Checklist-5” (PCL5) score, post-concussive symptoms using the “Neurobehavioral Symptom Inventory” (NSI) score, clinician referral and medical history. (i) Participants with post-concussive symptoms, clinician referral, history of medically documented mTBI, and scores ≥ 38 on the PCL5 and ≥ 26 on the NSI were grouped as the comorbid PCS + PTSD group. (ii) Participants with no history of mTBI in the last 5 years, a score ≥ 38 on PCL5, and < 26 on NSI and clinician referral were grouped as PTSD. (iii) Participants with score < 38 on PCL5 and < 26 on NSI, no mTBI within the last 5 years, no DSM-IV-TR or DSM-V diagnosis of a psychiatric disorder (based on medical records), and no history of moderate-to-severe TBI were grouped as combat controls. All participants were assessed by a licensed medical practitioner, and reported being deployed to a combat environment. Those with psychotic, mood or substance dependency disorders were excluded.

### Measures

A battery of psychological health measures were administered to the participants prior to their MRI scan. The battery consisted of the Brief Traumatic Brain Injury Screen [BTBIS; (Schwab et al., 2007)], PCL-5 (Blevins et al., 2015), NSI (Cicerone and Kalmar, 1995), Life Events Checklist (LEC; (Gray et al., 2004)), Combat Exposure Scale [CES; (Guyker et al., 2013)], Childhood Environment [CE; (King et al., 2003)], Zung Depression Scale [ZDS; (Zung et al., 1965)], Zung Anxiety Scale [ZAS; (Zung, 1971)], Alcohol Use Dependency Identification Test [AUDIT; (Saunders et al., 1993)], and the Epworth Sleepiness Scale [ESS; (Johns, 1991)]. In Supplementary Section “Psychological Health Measures,” (SI-2.1) we present, in more detail, the measures that were most relevant for this study [PCL5, NSI, and CNS vital signs (CNS-VS]). Neurocognitive composite index (NCI) was derived from CNS-VS domain scores (Gualtieri and Johnson, 2006) as an aggregate measure of neurocognitive functioning.

### Procedures

For procedures done prior to the fMRI scans during the scheduled appointment, see Supplementary Section “Procedures” (SI-2.2).

#### fMRI

Participants were scanned in a 3T MAGNETOM Verio scanner (Siemens Healthcare, Erlangen, Germany) using T2^*^ weighted multiband echo-planar imaging (EPI) sequence in resting state (participants would keep their eyes open and fixated on a white cross displayed on a dark background on the screen using an Avotec projection system, and not think of anything specific), with TR = 600 ms, TE = 30 ms, FA = 55°, slice gap = 1 mm, multiband factor = 2, anterior to posterior phase encoding direction, voxel size = 3 mm × 3 mm × 4 mm, and 1000 volumes. Brain coverage was confined to the cerebral cortex, subcortical structures, midbrain and pons (cerebellum was excluded). Two identical but separate scans were performed for every participant and processed independently [more information in the Supplementary Section “Procedures” (SI-2.2)].

### fMRI Data Pre-processing

Standard resting-state fMRI data pre-processing steps were done including realignment, normalization to MNI space, detrending and regressing out nuisance covariates such as six head-motion parameters, white matter signal and cerebrospinal fluid signal, and band-pass filtering (0.01–0.1 Hz). The largest permitted head motion was half the voxel-size (1.5 mm); no significant group differences were observed in participant head-motion (p > 0.05) (also see Table 1). Pre-processing was performed using Data Processing Assistant for Resting-State fMRI (DPARSF v1.7) (Chao-Gan and Yu-Feng, 2010), which is based on Statistical Parametric Mapping (SPM8) (Friston et al., 2007) and Resting-State fMRI Data Analysis Toolkit (Song et al., 2011).

**TABLE 1:** Mean, median and standard deviation of demographics, head motion, psychological measures (PCL5, NSI, and CES), and CNS-VS neurocognitive measures for each of the groups.

**Variable**		**Controls**	**PTSD**	**PCS + PTSD**
**DEMOGRAPHICS AND HEAD MOTION**				
Age, years	Mean	32.6	32.2	33.7
	Median	31	32	33
	SD	6.7	7.6	6.8
Education, years	Mean	15.1	14.5	14.1
	Median	16	14	14
	SD	1.9	2.2	1.9
Race	White	18 (66.7%)	11 (64.7%)	26 (66.7%)
	Black	2 (7.4%)	3 (17.6%)	9 (22.0%)
	Hispanic	3 (11.1%)	3 (17.6%)	2 (4.9%)
	Asian	2 (7.4%)	0	1 (2.4%)
	Other	0	0	1 (2.4%)
Head motion (mean frame-wise displacement)	Mean	0.098	0.121	0.111
	Median	0.072	0.076	0.069
	SD	0.082	0.106	0.104
Medication		2 (7.4%)	4 (23.5%)	13 (31.7%)^*^
Lifetime mTBIs	Mean (Range)	0.3 (2)	1.1 (6)	2.5 (15)^*^
**PSYCHOLOGICAL MEASURES**				
Traumatic stress^a^	Mean	23.5	56.6	70.9
	Median	21.5	48.5	70.5
	SD	4.2	17.8	15.2
Post-concussive symptoms^a^	Mean	6.6	25.9	43.4
	Median	5	17.5	41.5
	SD	4.8	19.2	16.1
Combat exposure^a^	Mean	7.2	16.7	28.6
	Median	2.5	15	29
	SD	9.8	11.2	8.6
**NEUROCOGNITIVE MEASURES**				
Neurocognitive composite index^t,z^	Mean	101.2	94.3	81.7
	Median	100.7	94.6	82.2
	SD	12.9	12.5	20.7
Reaction time	Mean	97.4	95.3	84
	Median	101	92	91
	SD	23	11.9	32.8
Complex attention^t^	Mean	94.2	78.1	70
	Median	99.5	92	80
	SD	23.3	30.9	31.3
Cognitive flexibility^t,z^	Mean	103.6	97.1	80.5
	Median	103	93	86
	SD	16.3	15.2	26.7
Processing speed^t^	Mean	104.8	100.1	89.9
	Median	104	98	92
	SD	20.9	11	20.1
Executive functioning^t,z^	Mean	106	101	84.1
	Median	104.5	104	90
	SD	13.3	13.2	24.8
Verbal memory	Mean	99.6	92.1	83.6
	Median	106.5	103	83
	SD	12.5	9.5	13.9

*^*a*^denotes *p* < 0.05, all three groups; ^*t*^denotes *p* < 0.05, Controls vs. PCS + PTSD, ^*z*^denotes *p* < 0.05, PTSD vs. PCS + PTSD. Traumatic Stress = PCL5; Post-concussive Symptoms = NSI; Combat Exposure = CES.*

**FIGURE 2:**
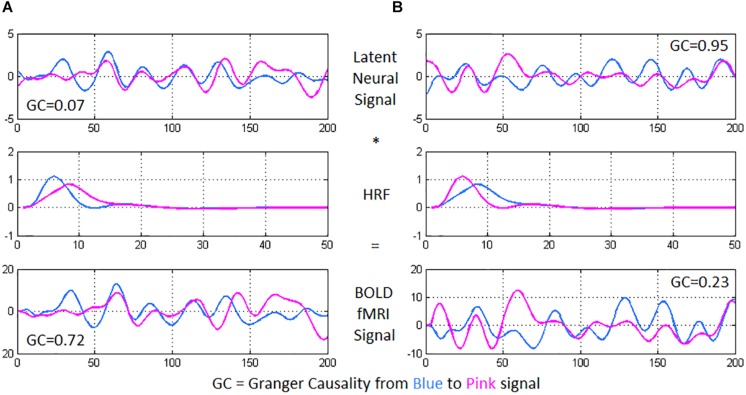
Using two time series from experimental fMRI data, we illustrate the importance of performing hemodynamic deconvolution for effective connectivity modeling. The latent neural signals are convolved with the hemodynamic response function (HRF) to provide the BOLD fMRI time series. Within-subject spatial HRF variability across the brain could often result in a scenario wherein **(A)** the latent neural signals have true low directional connectivity [quantified using Granger causality (GC) from blue to pink signal] while the BOLD fMRI time series show high GC wherein the pink time series seems to follow after the blue time series, and **(B)** the latent neural variables have true high directional connectivity while the BOLD fMRI time series show low GC. In the former case, while the neural signals nearly overlap, the delay in the HRFs causes an observable delay in the BOLD time series, resulting in high GC from the blue to the pink BOLD time series. In the latter case, the delay noticeable in the neural signals (pink signal leads blue) is negated by the delay in the HRFs (blue signal leads pink), resulting in nearly overlapping BOLD time series and a low GC value.

Deconvolution was then carried out on voxel-level time series, because confounds arising from spatial and inter-subject variability of the hemodynamic response function (HRF) (Handwerker et al., 2004; Rangaprakash et al., 2017c) could lead to a scenario wherein two fMRI time series show high effective connectivity but the underlying neural signals are not highly connected, and vice versa (refer to Figure 2 for an illustration) (Rangaprakash et al., 2018b, c). Such phenomena have been specifically found in the case of PTSD and mTBI with functional connectivity (Rangaprakash et al., 2017c). Additionally, causal connections could potentially switch directions in case the underlying HRFs possess different times-to-peak. In this respect, it has been demonstrated that deconvolution results in improved estimation of effective connectivity (David et al., 2008; Ryali et al., 2012, 2016). The viewpoint of cellular neuroscience on BOLD fMRI presented in a recent paper (Hall et al., 2016) discussed several caveats in the interpretation of fMRI results, wherein careful consideration is warranted based on the underlying cellular mechanisms. Neurovascular dynamics or HRF variability is one such primary issue, about which they comment as follows: “*advances in cellular neuroscience demonstrating differences in this neurovascular relationship in different brain regions, conditions or pathologies are often not accounted for when interpreting BOLD*.” They advise employing computational modeling (e.g., deconvolution) to mitigate the issue. We employed a popular blind deconvolution algorithm (Wu et al., 2013). Many recent papers have employed it [see for example (Amico et al., 2014; Lamichhane et al., 2014; Boly et al., 2015; Rangaprakash et al., 2017a, b)]. The deconvolution is blind since both the HRF and underlying latent neural time series are estimated only from the recorded fMRI data. Resting-state fMRI data is modeled as event-related using point processes with randomly occurring events; then, voxel-specific HRFs are estimated using Wiener deconvolution. This technique is date-driven; hence, we do not encounter overfitting issues that often plague model-based approaches.

Since whole-brain fMRI data has high dimensionality, 125 functionally homogeneous brain regions spread out across the cerebral cortex and encompassing it completely, determined using spectral clustering [known as the Craddock-200 atlas (Craddock et al., 2012)], were taken and mean deconvolved time series were obtained from them. All further analyses (carried out on the Matlab^®^ platform) utilized these 125 time series from every participant.

### Effective Connectivity Analysis

A precursor to obtaining network-level characterization is to first get the connectivity network itself, which is, computing SEC and DEC matrices from pre-processed fMRI data. Whole-brain SEC and DEC were computed using GC (Deshpande et al., 2010b), which is an exploratory technique used to quantitatively measure directional relationships between brain regions. While SEC uses a multivariate autoregressive (MVAR) model, DEC employs a dynamic MVAR model evaluated in a Kalman filter framework using variable parameter regression (Wheelock et al., 2014).

The concept of Granger causality (GC) is that, if future values of time series “Y2” can, in a mathematical sense, be predicted by the past values of time series “Y1,” then a causal influence is inferred from time series Y1 to time series Y2 (Granger, 1969). GC’s MVAR model predicts one time series from the other quantitatively, as described briefly next. Given *k* different time series *Z*(*t*) = [*z*_1_(*t*), *z*_2_(*t*), … *z*_*k*_(*t*)], wherein *k* corresponds to 125 ROIs of this work, the MVAR model of order *p* is given by:

(1) Z⁢(t)=M⁢(0)⁢Z⁢(t)+M⁢(1)⁢Z⁢(t-1)+M⁢(2)⁢Z⁢(t-2)+⋯+M⁢(p)⁢Z⁢(t-p)+E⁢(t)

Here, *E*(t) is the model error, while *M*(*0*) … *M*(*p*) are model coefficients. Like in earlier studies (Deshpande et al., 2010b), this formulation included a zero-lag term with coefficient *M*(*0*) which would eliminate the contribution of zero-lag cross-correlation between the time series. Since *M*(*0*) represents co-variance between time series and not used in GC computation, the effect of zero-lag cross-correlation gets ignored in GC. Given that diagonal elements of *M*(*0*) are set to zero, we only model the instantaneous cross-correlation, and not the auto-correlation between the time series.

The coefficients were estimated using multivariate least-squares estimation. It computes the set of optimal coefficients with model error being minimized in a least-squares sense. The model order p can either be chosen by utilizing a mathematical principle such as the Bayesian Information Criterion (BIC) (Roebroeck et al., 2005) or based upon the needs of the application being considered. In neuroimaging, causal relationships corresponding to neural delays less than or equal to the TR are of interest (Deshpande et al., 2013), hence we used a first order model. Given that fMRI’s temporal resolution is relatively low, a first-order model has been shown to capture the most relevant directional information (Deshpande and Hu, 2012).

The degree to which the past *Z*(*t-p*) is able to predict the present *Z*(*t*) is given by the coefficient matrix *M*(*p*). The sum of all such coefficients would then correspond to the degree to which the past values put together can predict the present. As in prior works (Kaminski et al., 2001), GC was formally derived, based on the model coefficients, as:

(2) $$G\,C_{i\,j}=\sum_{n=1}^{p}m_{i\,j}\,\left(n\right)$$

Wherein *m*_*ij*_ are the elements of matrix *M* and GC*_*ij*_* refers to the SEC value from ROI i to ROI j. Notably, a single coefficient matrix was computed for the entire duration of the experiment, that is, coefficients are not varying with time. A deeper theoretical rendering of GC can be found here (Deshpande et al., 2010b). GC-based methods have been experimentally validated for fMRI EC analysis (David et al., 2008; Katwal et al., 2013), and they have been extensively utilized for fMRI EC modeling in recent times [see for example (Deshpande et al., 2011, 2015)].

Next, DEC was computed by employing time-varying dynamic Granger causality (DGC), evaluated using a Kalman filter framework. Dynamic multivariate vector autoregressive (dMVAR) model was employed for estimating DGC (Grant et al., 2014; Wheelock et al., 2014). DEC is the underlying time-varying physiological process, while DGC is the mathematical measure that quantifies it. This technique has also been used in several recent studies (Deshpande et al., 2013; Wheelock et al., 2014; Hutcheson et al., 2015). Unlike GC formulation, dMVAR model coefficients *M’*(*p,t*) are a function of time, hence the model is “dynamic.”

(3) $$Z\,\left(t\right)=M^{\prime }\,\left(0,t\right)\,Z\,\left(t\right)+M^{\prime }\,\left(1,t\right)\,Z\,\left(t-1\right)+\cdots +M^{\prime }\,\left(p,t\right)\,Z\,\left(t-p\right)+E\,\left(t\right)$$

A Kalman filter framework which uses variable parameter regression (Büchel and Friston, 1998) was used to estimate dynamic model coefficients, which involved imposing a forgetting factor (which was chosen as 1 in our case). DGC was thus computed as:

(4) $$D\,G\,C_{i\,j}\,\left(t\right)=\sum_{n=1}^{p}m_{i\,j}\,\left(n,t\right)$$

Where *m*_*ij*_ are the elements of matrix *M* and *DGC*_*ij*_(*t*) is the value of DEC from ROI *i* to ROI *j* at a given time point *t*. Like in GC, zero-lag cross-correlation effects were compensated here also. Further, a forgetting factor of 1 was used to make the system well-conditioned so that the coefficients may be estimated accurately.

**FIGURE 3:**
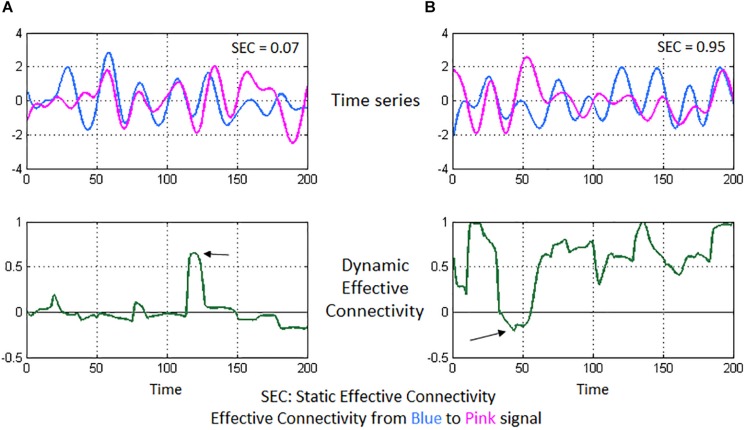
Illustration of static and dynamic effective connectivity (SEC and DEC) from a neuroimaging standpoint using two experimental fMRI time series. In **(A)**, the two time series seem highly correlated and nearly overlapping. However, the variations in the pink time series do not appear to happen after (or before) the variations in blue time series (top-left figure). This poor causal relationship results in a low SEC value (= 0.07). Correspondingly, DEC values hover around the zero-value (bottom-left figure) since a causal relationship does not seem to emerge for most part of time, except for a brief span (marked by the arrow) when there is a visible causal relationship. In **(B)**, the pink time series seems to constantly follow after the blue time series (top-right figure), indicating that the pink signal’s associated brain region activates (and deactivates) immediately after the blue signal’s region activates (and deactivates), thus a causal relationship and a high SEC value (= 0.95). DEC provides additional insight (bottom-right figure), wherein steady causality is maintained almost for the entire duration except for a brief span (marked by the arrow), wherein DEC dips because of observable lack of causality in the time series’ of those sections.

A 125 × 125 whole-brain SEC matrix was obtained for every participant by computing GC between all combinations of connections between the 125 ROIs. With DEC, the dynamic MVAR model coefficients are a function of time, hence, with our fMRI data having 1000 time points, we obtained a 125 × 125 × 1000 DEC matrix per participant. SEC and DEC matrices were used for further complex-network analysis. To illustrate the concepts of SEC and DEC in the context of neuroimaging, we show a simple illustration using a pair of fMRI time series from our experimental data (see Figure 3).

### Complex-Network Analysis

We first describe the network measures of segregation and integration, and then explain how they were used in the context of this work. As noted earlier, given the complexity of our hypothesis, we dealt with weighted directed networks in this work. Functional segregation was quantified using transitivity (global measure, one value for whole brain per participant), clustering coefficient and local efficiency (both local measures, one value per node/region per participant). Functional integration was quantified using global efficiency (global measure), shortest path length and edge betweenness (both local measures, one value per connection per participant). We obtained source codes for these measures from the Brain Connectivity Toolbox (April 2014 release) (Rubinov and Sporns, 2010), and implemented the entire pipeline in the Matlab^®^ platform through custom codes. A detailed account of these measures can be found in Rubinov and Sporns (2010). For the benefit of readers, we have explained each of these measures in detail using a simplified example network in Supplementary Section “Complex-Network Analysis” (SI-2.3).

Briefly, transitivity is a global measure of overall efficiency of local processing in the brain. Clustering coefficient (*CC*) gives a transitivity-type characterization for every node. Local efficiency (*EffLoc*) is closely related to *CC*, wherein nodes with powerful neighbors that are involved in several shortest paths have higher *EffLoc*, indicating that the node is important in the sub-network for specialized processing. While *CC* and *EffLoc* usually give similar (but not same) results, their interpretations are different. In this work, along with transitivity as the global measure, we employed both *CC* and *EffLoc* as local measures, which are the two popularly used local measures of segregation. We took an overlap (intersection) of the final significant group differences for the two measures, so that the affected nodes had differences in both the measures, thus providing more conservative results with a broader interpretation.

Global efficiency (*EffGlob*) is a global measure indicating the aggregate ease of communication in the entire network. Shortest path length (SPL) is a measure of how easy it is to reach one node from the other, and is analogous to meta-connectivity. Edge betweenness (*EB*) measures the number of all shortest paths in the entire network that contain the given connection. Like SPL, *EB* is an important network measure because it characterizes the importance of a connectivity path not only through its pairwise connectivity value but also through the significance of the connectivity path for other connectivity paths present in the network. If a connectivity path matters a lot for other paths, i.e., for communication between various other nodes, then the given path would have high integration ability (i.e., *SPL* and EB). Such a characterization can be obtained only through complex-network modeling since traditional pairwise connectivity informs us only about the strength of interaction between just two brain regions. In this work, we employed *EffGlob* as the global measure and both *SPL* and *EB* as local measures of integration. As with segregation, we took an overlap (intersection) of the final significant group differences in the two local measures, so that the affected paths had differences in both measures, thus providing more conservative, but potentially more reliable, results with wider interpretation.

Next, we describe how these six network measures were used in the context of this work. SEC and DEC connectivities were used separately to construct static and time-varying networks with brain regions as nodes and connectivity strengths between them as the weighted directed edges of the network graphs. Absolute value of connectivity was used to construct the network graphs. With SEC, a single network was constructed for the entire duration of time in the data, giving a “connectivity strength” network, which was used to obtain each one of the six complex-network measures for every run of every participant. With DEC, we considered each time point of the DEC time series as a snapshot of the network at that time instant, and then constructed a graph using the nodes and edge values from that snapshot. We computed network measures for that snapshot, and repeated the procedure independently for the rest of DEC time series to obtain a time series of values for each network measure. Then, for each network measure, we computed the variance of the network measure time series to obtain a single value for the entire duration of the data. This gave us a network with paths whose weights corresponded to the temporal variability of complex-network measures. This was obtained for every measure and for every run of every participant, similar to SEC.

Statistically significant differences in these strength and variability networks were obtained, in accordance with our hypothesis (*p* < 0.05, FDR corrected). We corrected for 31250 comparisons: 125 comparisons of segregation (125 ROIs), 15500 comparisons of integration (125 × 125 − 125), each for both static connectivity and variance of dynamic connectivity networks. Differences were controlled for age, race, education, and head-motion [using mean frame-wise displacement, as defined by Power et al. (2012)]. That is, we found significant group differences with both SEC and DEC derived complex-network measures separately for these three pairwise comparisons (thus giving a total of six comparisons per network measure): Control vs PTSD, Control vs. PCS + PTSD, PTSD vs. PCS + PTSD. We then identified the common network measures among four of these comparisons (*hypothesis-1*) which excluded PTSD vs PCS + PTSD comparison, and we also identified common network measures among all the six comparisons (intersection, hypothesis-2), all of which also fit our hypothesis, that is, conformed to the increasing/decreasing trend as we moved from Control to PTSD to PCS + PTSD.

It is notable that we have taken a conservative approach in this work. We opted to look for common differences in pairwise statistical comparisons, rather than performing a single three-way statistical comparison, which is less conservative. We obtained common differences in static as well as dynamic network measures, and we also constrained the differences to conform to a trend as per our hypothesis. Additionally, we computed two local measures in segregation as well as integration, and considered only common differences in them, which added another level of constraints on our findings. In addition to these, we notably discarded any paths which had significant network-level differences in local measures of integration (i.e., SPL and EB), but not significant pairwise effective connectivity differences themselves. That is, we included only those paths which had significantly different SEC and variance of DEC in accordance with the trend set out in our hypothesis (p < 0.05, FDR corrected, controlled for age, race, education and head-motion), in addition to having significantly different local measures of integration (i.e., SPL and EB). This was done to ensure that, irrespective of network-level disturbance, the significant connections that emerged in this work would have also cleared whole-brain multiple-comparisons-corrected statistical threshold with traditional static and dynamic effective connectivity like in most other studies. This reassured that our results conformed to multiple layers of validation, verification and statistical standards, and that evidence of network disruption were obtained via multiple analysis approaches, in addition to providing novel insights through network characterization.

### Behavioral Relevance of Network Properties

In an effort to assess the behavioral relevance of complex-network measures, we first obtained the association of the strength and variability of complex-network measures (only those which fit our hypothesis) with symptom severity in PCS (NSI score) and PTSD (PCL5 score), as well as neurocognitive functioning (NCI score and subtests). Neurocognitive functioning (e.g., executive functioning, cognitive flexibility) is often impaired in psychiatric disorders such as PTSD and PCS (Simmons and Matthews, 2012), hence identifying such network properties associated with it would be important. We report significant associations between complex-network properties and behavioral/clinical measures.

In order to obtain additional insight into how network properties of the ensemble of identified connections mapped on to the ensemble of behaviors, we performed partial least squares regression (PLSR) analysis (Krishnan et al., 2011), which we employed to predict neurocognitive functioning (NCI and subtests) and symptom severity (PCL5, NSI) from strength and variability of network measures obtained from our prior analysis. We present the percentage variance in behaviors explained by the complex-network measures.

### Machine Learning Classification Analysis

For predicting the diagnostic membership of a novel subject based on a novel measurement using the measure, success in hypothesis testing is neither necessary nor sufficient. A mechanism to quantify the predictive ability of the features is not available with the hypothesis testing framework, requiring us to acknowledge what a technique like hypothesis testing can do, and cannot do. Statistically significant network properties necessarily need not have high predictive ability, and vice versa. Hence, those network properties that are both statistically significant (in accordance with our hypothesis) and are top-classifiers (high predictive ability) carry superior importance and relevance. Therefore, we used machine learning techniques to identify such network properties (features) which can accurately classify individuals between controls, PTSD, and PCS + PTSD. A Recursive Cluster Elimination based Support Vector Machine (RCE-SVM) classifier (Deshpande et al., 2010a) was used to classify the participants based on whole-brain network properties (both strength and variability). Notably, findings from prior complex-network analysis were not used to bias the machine learning analysis as whole-brain data was used. A detailed account of this technique can be found in Rangaprakash et al. (2017a), and we have explained it thoroughly in Supplementary Section “Machine Learning Classification Analysis” (SI-2.4) to benefit the reader.

Briefly, RCE-SVM iteratively eliminates features to minimize prediction error. The training data is clustered, and upon SVM classification the clusters are scored using testing data. Low-scoring clusters are eliminated (*RCE step*) and the procedure is repeated until only the top-predictive features remain. In this work, we made the following parameter choices. The training set consisted of 80% of the participants, while the testing set consisted of the remaining 20%. We began the algorithm with forty clusters in the first RCE step. Based on performance, the bottom 20% of the clusters were eliminated in every subsequent RCE step. Two clusters containing the top-predictive features remained in the final RCE step. With a hundred random iterations, sixfold cross validation was performed in every iteration, resulting in a total of 600 iterations over the complete execution.

To be conservative, we obtained the worst-case classification accuracy by evaluating the lowest accuracy value gathered from test data among all 600 iterations (sixfolds × 100 repetitions). Statistical significance of the accuracies was computed through estimating *p*-values using a binomial null distribution B(η,ρ), with ρ being the probability of accurate classification and η being the number of participants like in previous studies (Pereira et al., 2009). Only accuracies with *p* < 0.05 (Bonferroni corrected) were taken as statistically significant.

We repeated this procedure and performed classification independently with 32 available non-imaging measures as input features instead of network measures. The 32 measures were: (i) psychological health measures: Perceived Stress Scale, Epworth Sleepiness Scale, Pittsburgh Sleep Quality Index, Zung Depression Scale, and Zung Anxiety Scale; (ii) behavioral measures: all CNS-VS measures including the NCI score; (iii) exposure/injury descriptives: CES, lifetime concussions, and Life Events Checklist. Worst-case accuracies and top-classifying features were obtained, with them being compared with the results obtained by using complex-network measures.

### Machine Learning Regression (Dimensional) Analysis

Network properties having statistical significance in accordance with our hypothesis, having behavioral relevance as well as having high predictive ability were attributed distinctive importance in this study. Using such features, we finally performed support vector regression (SVR) to predict PTSD and PCS symptom severity, in order to assess those features dimensionally. Similar to the classification analysis, we performed sixfold cross-validated linear SVR over one million iterations. Specifically, in each iteration, the regression model was developed using 5/6th of the randomly chosen participants. The model used features described above as inputs and learned the underlying function which maps onto the PCL5 and NSI scores. Subsequently, the model was used to predict PCL5 and NSI scores in the remaining 1/6th participants. Our machine learning classification and regression analyses involved no hyperparameter optimization. We report correlation (*R*^2^) between predicted and measured symptom severity scores.

**FIGURE 4:**
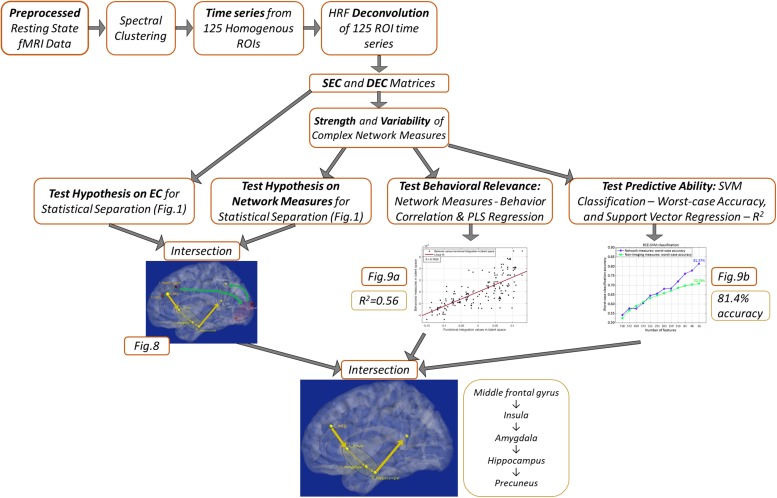
Schematic of the complete processing pipeline executed in this study. Results corresponding to different analyses are summarized, which can be viewed in more detail by referring to the corresponding figures.

Figure 4 summarizes the processing pipeline of all the methods.

## Results

### Demographics

The demographics (for the three groups) are presented in Table 1. There were no significant group differences in age, *p* = 0.70, or education, *p* = 0.15. The results indicated a difference in the frequency of reported psychotropic use between the groups, τb = 0.24, *p* = 0.01, with the highest percentage of medicated participants being in the comorbid group. The number of reported lifetime mTBIs also had significant group differences specifically between control group and the PCS + PTSD group [*F*(2,171) = 5.81, *p* = 0.004], but not the control versus PTSD groups or PTSD versus PCS + PTSD groups, *p* > 0.05.

### Psychological Health and Neurocognitive Function

The results revealed significant differences between the three groups in posttraumatic symptoms (PCL5), [*F*(2,81) = 101.65, *p* < 0.001], post-concussive symptoms (NSI), [*F*(2,78) = 49.79, *p* < 0.001], and CES, *F*(2,79) = 40.69, *p* < 0.001. All *p*-values remained significant after corrections for multiple comparisons. As observed in Table 1, the PCS + PTSD group had the highest scores out of the three groups on these respective measures.

The results indicated that, after corrections for multiple comparisons, the control group displayed significantly higher scores than the PCS + PTSD group on all neurocognitive measures, *p* < 0.05, except for reaction time and verbal memory, *p* > 0.05. The PCS + PTSD group also had significantly lower scores in executive functioning, cognitive flexibility, and the NCI compared to the PTSD group, *p* < 0.05. The findings suggest that both the PTSD and PCS + PTSD groups display lower scores than controls, but also, the comorbid group had greater impairments than the PTSD group (see Table 1).

### Complex-Network Analysis Using Effective Connectivity

**FIGURE 5:**
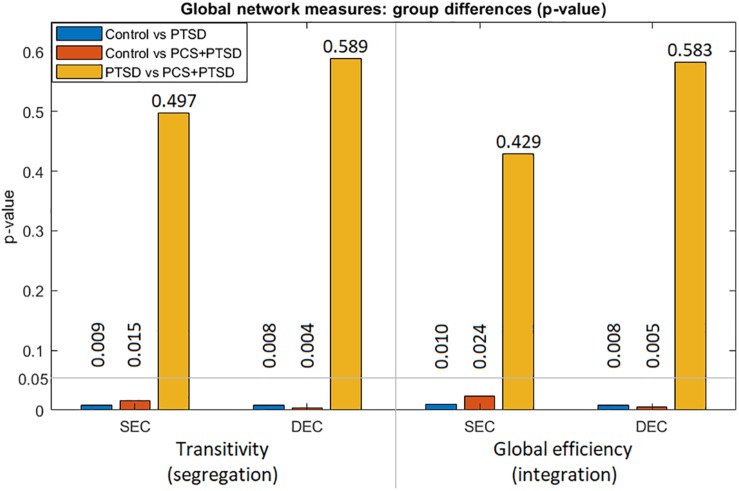
Group differences (*p*-values) for the two global measures (transitivity and global efficiency) obtained from both SEC and DEC matrices. We observe that whole-brain differences were driven by PTSD, while mTBI likely did not cause whole-brain-level changes.

We used SEC and DEC connectivity matrices to compute six complex-network measures (two global and four local measures). With global measures (Figure 5), we found significantly lower strength and variability of both segregation and integration in PTSD and PCS + PTSD compared to controls. Our finding indicates that both specialized processing and efficient communication are compromised in the disorders at the whole brain level. However, no significant differences were found between PTSD and PCS + PTSD groups, indicating that PTSD might contribute to global aberrations whereas the effect of mTBI might be more localized.

#### Local Measures

**FIGURE 6:**
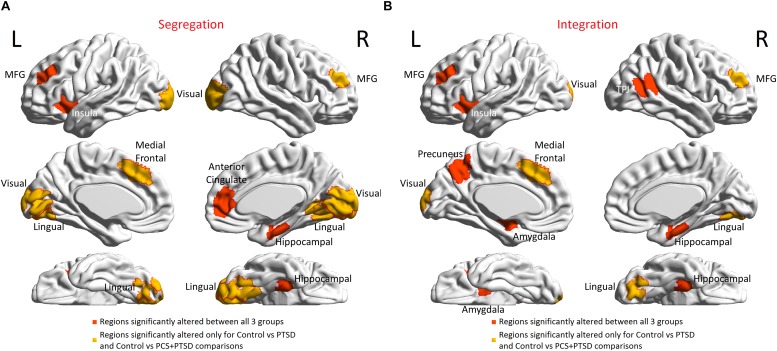
Brain regions with altered functional segregation and/or integration across groups. **(A)** Regions-of-interest (ROIs) associated with significantly disrupted functional segregation. **(B)** ROIs associated with significantly disrupted functional integration. The ROIs were defined by the Craddock-200 atlas (Craddock et al., 2012). MFG, middle frontal gyrus; TPJ, temporo-parietal junction.

Further granularity was obtained with local measures. Altered segregation was mainly observed in prefrontal and occipital regions (Figure 6A). None of the occipital regions were statistically different between the PTSD and PCS + PTSD groups, while the majority of the remaining identified regions were significantly different. While these results were obtained using a strict statistical threshold, we noticed that when a liberal threshold was used (not shown here), more prefrontal nodes were affected compared to parietal/occipital nodes, which were all characterized by lower segregation. This might explain why we observed lower transitivity (global segregation) in PTSD and PCS + PTSD compared to controls.

Generally, the terms “*inflation*” and “*deflation*” (Figure 1) correspond to elevation (increased value) or suppression (decreased value) of static network properties, respectively, and not just connection strengths of individual paths. Similarly, “rigidity” corresponds to lower temporal variability of dynamic network properties. However, in the special case when local network properties of paths (i.e., integration) were considered, these terms referred to network properties as well as connection strengths of the paths under consideration.

**FIGURE 7:**
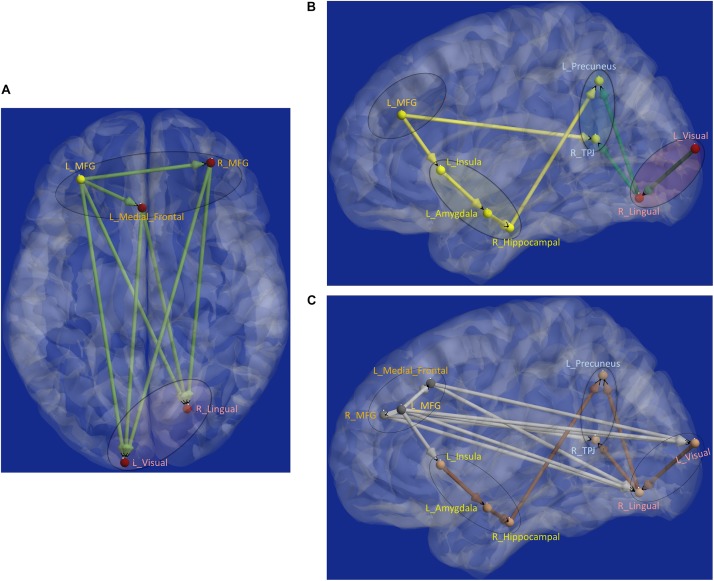
Functional segregation/integration (metastability) results. **(A)** The network of integration was broken down into two sub-networks. **(A)** Shows the first of the two sub-networks, the fronto-visual sub-network that exhibited lower strength of integration and lower temporal variation of integration, which was significant for control vs. PTSD and control vs. PCS + PTSD comparisons (but not PTSD vs. PCS + PTSD comparison). The yellow node had altered segregation between all three groups, while the red nodes were different except for the PTSD vs. PCS + PTSD comparison. This sub-network likely represents reduced prefrontal inhibition of visual memory processing and retrieval. **(B)** Second of the two sub-networks, the parietal *inflation* sub-network that exhibited altered strength of integration and lower temporal variation of integration. Yellow paths were significantly different for all group-wise comparisons. Green paths were altered except for the PTSD vs. PCS + PTSD comparison. This sub-network showed parietal-*inflation* caused by subcortical and visual network disruptions, which were in-turn driven by the left middle frontal gyrus (MFG). **(C)** The entire network of disruption found in the work, showing nodes/paths in gray which were on a rigid deflated regime (lower strength and temporal variability of segregation/integration; as well as lower strength and variability of effective connectivity, marking hypo-connected inflexible connectivity), and nodes/paths in brown, which were on a rigid *inflation* regime (higher strength of segregation/integration and lower variation of segregation/integration over time; as well as higher strength and lower variability of effective connectivity, marking hyper-connected inflexible connectivity). Noticeably, all prefrontal nodes and prefrontal-originating paths exhibited a deflated regime, while the rest (those not associated with prefrontal regions) exhibit an inflated regime. Such a lucid dichotomy is interesting. It is clearly observable that *deflation* originates in the prefrontal cortex, which subsequently results in the *inflation* of parietal regions through two routes, subcortical and visual. MFG, middle frontal gyrus; TPJ, temporo-parietal junction.

Next, aberrant local measures of integration were found along two distinct pathways (see Figure 6B for the affected ROIs), which we present as two subnetworks for clarity: (i) fronto-visual subnetwork (Figure 7A), and (ii) parietal-*inflation* subnetwork (Figure 7B). The fronto-visual subnetwork showed prefrontal *deflation* of secondary visual areas and lingual gyrus, i.e., lower strength/variance of network properties (SPL and EB) of paths connecting certain prefrontal regions to certain visual regions. This subnetwork was, however, not significantly different between the PTSD and PCS + PTSD groups, indicating that it might not be affected by an mTBI (since one difference between these groups is a history of significant prior mTBI(s) in the PCS + PTSD group). Notably, all paths here also exhibited lower SEC/vDEC connectivity values in addition to lower strength and variability of integration.

The parietal-*inflation* subnetwork (see Figure 7B) showed that the visual areas affected in the fronto-visual subnetwork were driving two key parietal regions [precuneus, temporo-parietal-junction (TPJ)]. Additionally, we observed fronto-subcortical disinhibition resulting in rigid *inflation* (increased strength but lower variance of network properties SPL and EB) of key subcortical areas (amygdala, hippocampus) and anterior insula, which subsequently resulted in the *inflation* of the same key parietal regions (precuneus, TPJ). Interestingly, this fronto-subcortical-parietal subnetwork was significantly different between all three groups, indicating that both PTSD and mTBI affect this subnetwork, while the occipital part was not significantly different between PTSD and PCS + PTSD (see Figure 7C). This is a potentially important finding.

**FIGURE 8:**
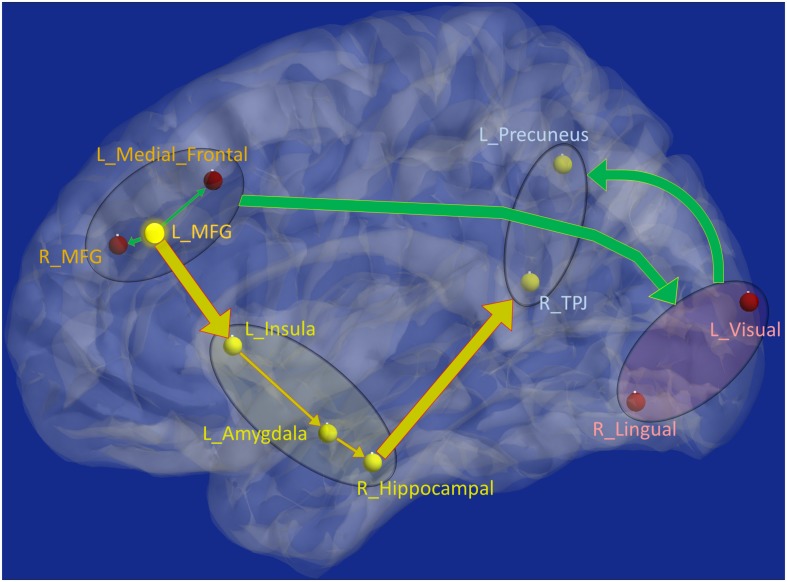
Schematic of the entire network: Yellow nodes/paths were significantly different for all three group-wise comparisons. Green paths (and red nodes) were significantly different except for the PTSD vs. PCS + PTSD comparison. Thick lines correspond to connections between major sub-networks while thin lines correspond to connections within sub-networks. The prefrontal sub-network consisted of MFG and medial frontal, the parietal sub-network consisted of TPJ and precuneus, the visual sub-network consisted of lingual and primary visual areas while the emotion-memory sub-network consisted of sub-cortical regions such as amygdala and hippocampus and cortical regions such as the insula. Disrupted left-MFG causes *deflation* of emotion-memory regions and visual memory-related regions, culminating in parietal-*inflation* causing heightened symptoms often observed in PTSD and PCS.

Schematic of the entire network (Figure 8) shows that the left middle frontal gyrus (MFG), which largely overlaps with the dorsolateral prefrontal cortex (DLPFC), is the likely source of the network-level disruption, whose *deflation* (suppressed network properties) results in *inflation* (elevated network properties) of downstream subcortical and visual pathways, culminating in parietal *inflation*. Figures 6–8 were visualized using BrainNet Viewer (Xia et al., 2013). In Supplementary Information, we provide observations from additional supplemental analysis performed by us (i) using a different brain parcellation instead of Craddock-200 [see Supplementary Section “Observations Using a Different Brain Parcellation Instead of Craddock-200” (SI-3.1)], (ii) using eigenvariate time series data instead of mean time series [see Supplementary Section “Observations Using Eigenvariate Time Series Data Instead of Mean Time Series” (SI-3.2)], and (iii) using ROI-level deconvolved data instead of voxel-level deconvolved data [see Supplementary Section “Observations Using ROI-Level Deconvolved Data Instead of Voxel-level Deconvolved Data” (SI-3.3)]. Our results remained consistent across different choices.

### Behavioral Relevance of Network Properties

Strength and temporal variability of functional integration values of four paths, which were significantly different between all three groups (the yellow connections in Figure 8), as well as the strength and temporal variability of functional segregation of MFG and Insula (Figure 6A) showed significant associations (*p* < 0.05 Bonferroni corrected) with neurocognitive functioning (NCI) and severity of both PTSD symptoms (PCL5-score) and post-concussive symptoms (NSI-score), thus highlighting their relevance to the underlying pathophysiology (see Table 2). Notably the associations followed the expected trend: increase in symptom severity and decrease in behavioral performance corresponded to higher strength of integration in inflated paths and lower in deflated paths, and lower variability (i.e., rigidity) in integration in all paths (similarly with segregation). However, those connections which were not different between PTSD and PCS + PTSD (green paths in Figure 8), as well as other nodes in Figure 6A and global complex-network measures had no significant associations with symptoms and neurocognitive performance.

**TABLE 2:** Association of strength and variability of complex-network measures with the NCI score and symptom severity in PTSD (PCL5 score) and PCS (NSI score).

**Complex network measure**	**Path (Integration) or node (Segregation)**	**Symptom severity score**		**Behavioral measure**
				***Neurocognitive***
		***PCL5 score***	***NSI score***	***Composite***
		***(PTSD)***	***(PCS)***	***Index (NCI)***
**Static functional integration measures**				
Shortest path length	L_MFG → L_Insula	–0.6902	–0.6756	0.6589
	L_Insula → L_Amygdala	0.6822	0.6759	–0.6298
	L_Amyg → R_Hippocampus	0.6535	0.6930	–0.6389
	R_Hippocampus → L_Precuneus	0.6990	0.6580	–0.3545
Edge betweenness	L_MFG → L_Insula	–0.6704	–0.6853	0.5871
	L_Insula → L_Amygdala	0.7370	0.6868	–0.5303
	L_Amyg → R_Hippocampus	0.7080	0.6372	–0.3956
	R_Hippocampus → L_Precuneus	0.7156	0.6669	–0.4193
**Variance of dynamic functional integration**				
Shortest path length	L_MFG → L_Insula	–0.7532	–0.7327	0.6704
	L_Insula → L_Amygdala	–0.7579	–0.7382	0.6748
	L_Amyg → R_Hippocampus	–0.7541	–0.7358	0.6709
	R_Hippocampus → L_Precuneus	–0.8520	–0.7737	0.4579
Edge betweenness	L_MFG → L_Insula	–0.7330	–0.7287	0.6672
	L_Insula → L_Amygdala	–0.7358	–0.7260	0.6586
	L_Amyg → R_Hippocampus	–0.7326	–0.7264	0.6590
	R_Hippocampus → L_Precuneus	–0.8513	–0.7776	0.4619
**Static functional segregation measures**				
Clustering Coefficient	L_MFG	–0.6859	–0.6685	0.6245
Local Efficiency	L_MFG	–0.7013	–0.6990	0.6826
	L_Insula	0.6527	0.6550	–0.6290
**Dynamic functional segregation measures**				
Clustering Coefficient	L_MFG	–0.7478	–0.7271	0.6538
	L_Insula	–0.7412	–0.7204	0.6533
Local Efficiency	L_MFG	–0.7524	–0.7324	0.6692

*Table presents the correlation values (*R*-value), which were significant with *p* < 0.05 Bonferroni corrected.*

**FIGURE 9:**
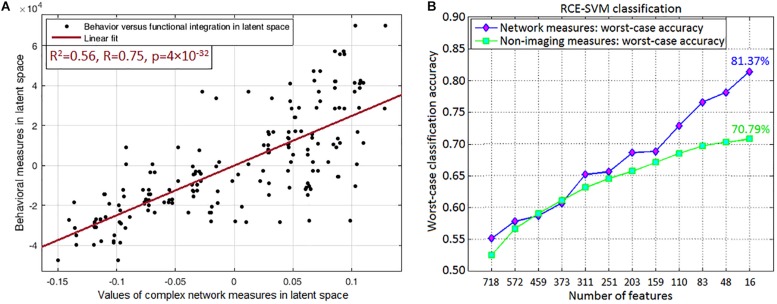
Association and prediction results. **(A)** Partial least squares regression maps independent (all included network measures combined) and dependent (all neurobehaviors combined) variables into a latent space to find an aggregate relationship between them. The regression displayed in the figure is performed in the latent space, which contains categorical variables representing all included network measures of the four integration paths (MFG → Insula, Insula → Amygdala, Amygdala → Hippocampus, Hippocampus → Precuneus) and two functional segregation nodes (MFG, Insula) (both of which fit our hypothesis), and the neurobehaviors in latent space. Their association in the latent space could be considered as the net association of all these network measures with all the neurobehaviors. **(A)** Shows the linear fit in latent space (*R*^2^ = 0.56, *R* = 0.75, *p* = 3.5 × 10^–32^). **(B)** Machine learning classification result, which was obtained through recursive cluster elimination based support vector machine (RCE-SVM) classifier, to classify between control, PTSD and PCS + PTSD groups. **(B)** Shows worst-case classification accuracies obtained using recursively reducing number of discriminative features (poorer features are successively eliminated). Classification was independently performed with both complex-network measures obtained from the entire brain and non-imaging measures (NIMs). We observed that network measures outperformed NIMs, with approximately 10% superior performance in the final RCE step using top-predictive features of network measures.

Since multitude of network paths and nodes had relevant associations with multiple measures of symptoms and neurocognitive performance (which we will now refer to as neurobehavioral indices), it would be interesting to measure how much variance in the neurobehavioral indices could be explained by those set of network measures. This was accomplished using PLSR (Krishnan et al., 2011), which finds the combined ability of the strength and variability of functional integration of the four connections and functional segregation of two nodes to predict neurobehavior. We found that the strength of network measures could explain 48.95% variance in the neurobehavioral indices, while the temporal variability of network measures could explain 57.17% variance. When both were combined, they could explain 61.74% variance in the neurobehavioral indices. A significantly large association between these network measures and neurobehavior (*R*^2^ = 0.56, *R* = 0.75, *p* = 3.5 × 10^–32^) was observed in the latent space (see Figure 9A for linear fit). The latent space consists of categorical variables that represent all network measures and all neurobehavioral indices included into the model, so that their relationship in the latent space could be considered the effective association of all the included network measures with all the neurobehaviors. As such, the latent space variables contain more “information” in them than the individual variables themselves, consequently explaining more variance than individual measures. For this reason, our finding of higher *R*^2^-value must not be surprising (Vul et al., 2009), and this is fundamental to the multivariate nature of PLS, as elaborated by Krishnan et al. (2011). Our finding reiterates that the strength and variability of functional integration of the four paths and that of segregation of the two nodes identified in this work are behaviorally relevant.

One a side note, head motion [mean frame-wise displacement (Power et al., 2012)] was not significantly correlated with behavioral measures in latent space (*R* = 0.049, *p* = 0.52), complex network measures in latent space (*R* = 0.056, *p* = 0.46), PCL5 (*R* = 0.047, *p* = 0.54) or NSI (*R* = 0.015, *p* = 0.84) symptom severity scores. This enhanced our confidence in the results.

### Machine Learning Classification Results

Top predictors are those that, among all network measures, possess the highest ability in predicting the diagnostic membership of a novel subject. To identify the top-predictors, we performed RCE-SVM classification (Deshpande et al., 2010a). Classification was done with two different paradigms: (i) classification using the 32 non-imaging measures (NIMs), and (ii) classification using strength and temporal variability of network measures taken from the entire brain (all data, nothing left out). Results showed that classification using network measures provided significantly better accuracy (approximately 10% more, *p* < 0.05 Bonferroni-corrected) than classification using NIMs (Figure 9B). This result indicates that network measures have superior predictive ability in identifying individuals with PCS and PTSD as compared to NIMs.

Table 3 shows the worst-case accuracies and top predictive features (for average accuracy, please see Supplementary Section “Supplemental Machine Learning Classification Results” (SI-3.4) and Supplementary Figure S3). Also of considerable interest are the top-predictors that resulted in highest classification accuracy. For classification using network measures, strength, and temporal variability of functional integration of the following four paths were the top predictive features: L_MFG → L_Insula, L_Insula → L_Amygdala, L_Amygdala → R_Hippocampus and R_Hippocampus → L_Precuneus). Coincidentally these four paths also showed statistically significant differences in static as well as time-varying network properties (the yellow paths in Figure 8, which were significantly different between all three groups). Also, coincidentally, these were the same four paths whose network measures had significant associations with neurocognitive functioning and symptom severity. To expand upon this, our findings revealed that, in addition to behavioral relevance and statistical separation, these paths also possessed the highest predictive ability, all obtained in a data-driven fashion from whole-brain complex-network data.

**TABLE 3:** Machine learning classification was performed using recursive cluster elimination based support vector machine (RCE-SVM), to classify between controls, PTSD and PCS + PTSD groups.

	**Worst-case**	
	**accuracy**	**Top-predictive features**
Non-imaging measures	70.79%	Epworth sleepiness scale and Zung depression scale
Complex network measures	81.37%	Strength and variability of functional integration of the four yellow paths in Figure 8
*p*-value for row-wise comparison	7.81 × 10^–28^	

*Table presents the obtained worst-case classification accuracies along with top-predictive features.*

### Machine Learning Regression Results

Finally, using these network properties (the yellow paths in Figure 8), we performed SVR to predict PCL5 (and NSI) scores. Over one million iterations, we found that predicted and measured PCL5 scores were significantly correlated (*R*^2^ = 0.72 ± 0.05, *R* = 0.85 ± 0.03, *P* = 3 × 10^–7^ ± 10^–6^), as also were predicted and measured NSI scores (*R*^2^ = 0.66 ± 0.04, *R* = 0.81 ± 0.03, *P* = 7 × 10^–7^ ± 10^–6^). With such high predictive ability (i.e., 66–72% variance explained in symptom severity), these network properties assume considerable importance in the context of PTSD and PCS + PTSD pathology. Figure 4 summarizes the processing pipeline of our entire work, along with corresponding results.

## Discussion

In the current study, we successfully employed a novel complex-network modeling framework to understand network-level impairments in PTSD with and without mTBI. With the evidence that the healthy brain is characterized by a balance between functional segregation and integration, we sought to identify aberrations in segregation and integration in these disorders. We hypothesized that *PTSD and mTBI are characterized by altered strength and lower temporal variability of segregation and integration in directional brain networks*. Specifically, we sought to identify networks that were affected by PTSD but not mTBI (hypothesis-1), as well as those affected by both PTSD and mTBI (PTSD and PCS + PTSD group) (hypothesis-2). We found evidence to support both hypotheses. This is the first fMRI study utilizing EC network modeling in either PTSD or PCS or the comorbid condition; the first study aiming to classify PTSD from comorbid PTSD/mTBI based on resting-state network properties using machine learning techniques; and the first study to examine network properties using both static and time-varying methods.

With global measures, we found that segregation and integration were significantly different for control vs PTSD and control vs PCS + PTSD comparisons only. This implies that the clinical groups had aberrations at the whole-brain level compared to controls, which is expected. However, the PTSD and comorbid groups do not exhibit any differences at the whole-brain level, suggesting that mTBI might result in more localized aberrations not detectable by network modeling at the whole-brain level. To further investigate discrete group differences, we used local measures of segregation and integration.

In accordance with our hypothesis (Figure 1), group differences in local segregation measures showed a clear dichotomy between prefrontal and occipital regions (Figure 6A); with all identified prefrontal nodes having lower segregation and all identified occipital and subcortical nodes having higher segregation. This indicates disruptive reduction in specialized local processing in the prefrontal cortex, especially in the MFG and medial prefrontal regions. This disruption had a negative relationship with the occipital and subcortical nodes, which showed disruptive increase in local processing. In addition, none of the occipital nodes were significantly different between PTSD and PCS + PTSD, implying that those regions might not differentiate between PTSD and PCS + PTSD.

With local integration measures, we found a clear dichotomy along two distinct pathways. The fronto-visual-parietal pathway (Figure 7A) was not significantly different between PTSD and PCS + PTSD groups, indicating that mTBI likely does not have a significant impact on this part of the network. Neither these paths (either connectivities or integration measures) nor the associated occipital nodes (segregation) exhibited any significant association with symptom severity (PCL5 and NSI) or neurocognitive functioning, hence we inferred that this part of the network does not play a significant role in symptom expression, but it might act as a supportive backend for other neural processes causing the symptoms. The other pathway (fronto-subcortical-parietal, Figure 7A) was significantly different between all the three groups, and network properties of the paths and associated nodes also showed significant associations with symptom severity and neurocognitive functioning. Thus, we inferred that disruption of this part of the network contributes to symptom expression, and is likely implicated in mTBI pathology. This dichotomy provides novel insights into our understanding of both common and distinguishing network characteristics in PTSD and mTBI, which has largely plagued the field, given the high comorbidity and overlapping symptomatology between them (Simmons and Matthews, 2012).

Another clear dichotomy arises in the strength of network properties across groups. All prefrontal nodes and the paths associated with them showed lower segregation/integration in PTSD and PCS + PTSD compared to controls, suggesting a strong effect of disruptive *deflation* prevalent in the prefrontal cortex. All the subcortical, parietal and occipital nodes showed higher segregation, and all paths associated with them not involving prefrontal regions showed higher integration in PTSD and PCS + PTSD compared to controls, a clear indication of disruptive *inflation* in these regions. Notably, these trends were also replicated in the raw effective connectivity values. We argue that this is definitive evidence for impaired prefrontal top-down regulation causing reduced control over limbic structures and other regions responsible for symptom-expression. Such unambiguous dichotomy clearly delineates the distinct functionality between the prefrontal cortex and the rest of the brain, and highlights its relevance to PTSD and mTBI.

Such dichotomy was not observed in the temporal variability of network properties, in that all nodes/paths showed lower variance, indicating some degree of pathological “frozen” state (in accordance with our hypothesis). In other words, paths with lower strength of network properties (*deflation*) tended to remain in that state over the duration of the scan, potentially suggesting impaired ability to increase the connectivity and the values of complex-network measures. Similarly, paths with higher strength of network properties (*inflation*) tended to remain inflated, also indicating impaired ability to decrease the connectivity and the values of complex-network measures. In total, we identified 15 nodes (segregation) and 16 paths (integration) which were significantly different with the control vs. PTSD and control vs PCS + PTSD comparisons, while only four nodes and five paths were significantly different between all the three groups. It is noteworthy that all the other nodes, with the exception of the amygdala and parietal regions, involved in the connections with affected functional integration also had altered segregation, implying segregation-integration imbalance in these regions. This observation corroborates with prior works, which have found evidence for a fine balance between segregation and integration (or metastability) in healthy individuals (Hellyer et al., 2015), which is disrupted in neurologic and psychiatric disorders (Yu et al., 2013; Rocca et al., 2014).

The networks were obtained with resting-state fMRI data; hence, they represent the differences in baseline state between the groups. Based on the prior knowledge regarding the neural mechanisms underlying cognitive emotion regulation (Gross, 2014), we propose that our network (Figure 8) corresponds to an aberrant emotion regulation system, with impaired prefrontal control leading to an insufficient control over emotionally intensive traumatic memories, which might underpin trauma re-experiencing, flashbacks, hyperarousal and other symptoms in soldiers with PTSD and PCS + PTSD.

Functions of the individual nodes/regions that were identified as having aberrations in the complex-network properties provides interesting insights into the neuropathology underpinning PTSD and mTBI. The MFG has been implicated in cognitive control (Emmert et al., 2016), which includes emotion regulation. It plays a pivotal role in the initiation of voluntary regulation of emotion (Gross, 2014). All of the network-level aberrations in our results could be traced back to the MFG (by tracing the directional connections), leading us to the conclusion that the MFG is the origin of network disruption in these disorders. Several earlier works have speculated about the MFG to be the likely origin of network disruption in PTSD (White et al., 2014; Kennis et al., 2015), including a recent meta-analysis (Simmons and Matthews, 2012). However, direct evidence for such a hypothesis has not been found so far. We provide novel evidence that supports this explanation. In fact, a recent meta-analysis presented evidence from numerous findings that repetitive transcranial magnetic stimulation (rTMS) applied to the MFG may be effective as a treatment for PTSD (Berlim and Van Den Eynde, 2014). Corroborating this, we discovered the network of disturbance caused by the impairment of MFG, wherein MFG is the source of disruptions. Taken together, the MFG likely plays a key role in the initiation of cognitive control necessary for emotion regulation, which when compromised, likely contributes to the maintenance of symptoms associated with PTSD and PCS + PTSD.

We noticed prefrontal top-down *deflation* of functional integration driven by the MFG, resulting in the *inflation* of functional integration in sub-cortical structures via the insula as well as parietal memory-related and sensory association regions. The anterior insula plays a major role in mediating prefrontal control over subcortical regions, and is thus found to be involved in emotion regulation and dysregulation (Thayer and Lane, 2000; Gross, 2014). It is structurally well connected with the amygdala through white-matter tracts (Oishi et al., 2015), and also plays a key role in subjective emotional experiences (feelings), integrating emotionally relevant information through multiple sources, and possibly representing them as one of the many complex emotions (Thayer and Lane, 2000). We found that prefrontal *deflation* of the insula causes inflated functional integration in the amygdala, which then results in inflated local functional integration in the hippocampus. *Inflation* of the hippocampus, a region crucial for declarative memories, might imply elevated explicit traumatic memory retrieval. It is well documented that both the hippocampus and the amygdala play a vital role in mTBI and PTSD (Simmons and Matthews, 2012; Costanzo et al., 2014). Since traumatic memories are unique in the intensity of associated negative emotions, emotion and memory share deep interconnection in PTSD (Vasterling et al., 2009).

The precuneus plays an important role in the generation of the experience of visual memories, whereas the TPJ is necessary for higher-level audio-visual verbalization and information processing (Gross, 2014). The path from the MFG leading to these regions was characterized by reduced strength and variance of functional integration. Thus, the memory-related and sensory association network comprising the precuneus and TPJ may translate to subcortical *inflation* and lack of prefrontal control, contributing to the perseveration of traumatic memories as observed in soldiers with PTSD.

There was a robust finding of disruption in the occipital regions in our results. While the majority of the nodes and paths were associated with the occipital region, none of them were significantly different between PTSD and PCS + PTSD groups, and none of them had behavioral relevance (through associations with symptom severity and neurocognitive performance). Hence, we inferred that this part of the network does not play a significant role in symptom generation, but might act as a supportive backend for the other fronto-subcortical-parietal processes, which do appear to contribute to the symptoms (owing to their association with symptom severity). This inference is justifiable, given that the visual imagery aspect of traumatic memories dominates the experience of vivid imagery associated with traumatic memory perseveration in PTSD (Hayes et al., 2012). It is known that the secondary visual regions, including the lingual gyrus, largely enables visual imagery (Thompson et al., 2009). In addition, the degree of activation in visual areas during imagery is directly proportional to the visual intensity of the object being imagined (Carpenter et al., 1999). Hence, it is likely that this measure is not sensitive to discriminate between PTSD and PCS + PTSD groups. This could provide substantiation for our inference that the occipital part of the network might be a backend process providing “imagery support.” Thus, it appears more likely that symptom severity can be attributed to the disruptions originating in the MFG, as illustrated by significant associations with neurocognitive performance and symptom scores.

There has been little success in addressing diagnostic limitations associated with homogeneity of symptoms and high comorbidity between PTSD and mTBI in military personnel (Costanzo et al., 2014). It is acknowledged that the additional burden of an mTBI in comorbid PCS + PTSD results in increased symptom severity (Vasterling et al., 2009). In the current study, we provide a mechanistic basis that might distinguish the underlying neurologic disruptions contributing to symptoms reported by PTSD cases from those reported by comorbid PTSD/mTBI cases. It is unclear as to whether the differences between the PTSD and PCS + PTSD groups are driven by higher symptom severity of the PCS + PTSD group or by impairments in white-matter integrity caused by an mTBI. A recent study found diffused white-matter tracts between the hippocampus and striatum to be the cause of corresponding functional connectivity differences between PTSD and comorbid PTSD/mTBI conditions (Rangaprakash et al., 2017a), yet it is unclear whether they can be extended to our findings. The network pathways that seem to differentiate, based on the strength of associations with neurocognitive measures, between the PTSD and comorbid PTSD/mTBI cases (PCS + PTSD) were the MFG, insula, amygdala, hippocampus, and precuneus.

**FIGURE 10:**
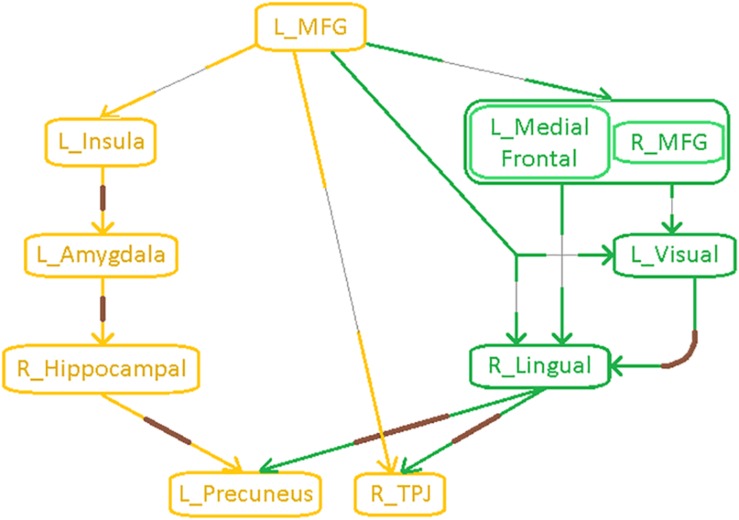
Flowchart illustrating an integrated model of connectivity and network-level aberrations in PTSD and mTBI. Paths with thin gray lines correspond to lower strength of network properties and connectivity (SEC) and lower temporal variability of network properties (i.e., rigidity) and connectivity (vDEC) in the clinical groups compared to healthy controls, indicative of breakdown in prefrontal top-down modulation. Paths with thick brown lines correspond to higher strength and lower temporal variability (i.e., rigidity) of network properties and connectivity, indicative of *inflation* in subcortical limbic and parietal memory-related regions. Yellow paths were significantly different between all the three groups, while the green paths were significantly different only for the control vs. PTSD and control vs. PCS + PTSD comparisons.

Our results are significant given that regions identified here have been implicated (albeit inconsistently) in earlier studies (Hayes et al., 2012; Simmons and Matthews, 2012; Eierud et al., 2014) to be involved in both PTSD and mTBI; however, a precise understanding of the underlying mechanisms, network structure, and their subsequent causal relationships has not emerged from them. With the help of a novel framework involving complex-network modeling with static and dynamic EC networks, we identified the nodes and network paths associated with the disorders, and detailed their directional relationships. We also highlighted the commonalities and differences in the PTSD and PCS + PTSD networks. Our characterization corroborates with behavioral manifestations of PTSD and PCS + PTSD, thus substantiating the utility and fidelity of our approach. Figure 10 summarizes our network-level findings with a flowchart.

Additionally, functional integration of four specific paths had significant associations with neurocognitive performance and symptom severity (MFG → insula, insula → amygdala, amygdala → hippocampus, hippocampus → precuneus), as also did functional segregation of two nodes (MFG and insula), highlighting their relevance to the underlying neurobehavior and symptomatology. These paths and nodes were the same as those which were identified as significantly different between all the three groups (Figure 8). In the PLS regression model, the aforementioned network measures explained approximately 62% variance in neurobehavioral measures.

Finally, we employed supervised machine learning classification to identify top predictors that could diagnose a novel subject. Literature is highly limited on the application of machine learning to the classification of either PTSD or mTBI [see notable recent works (Liu et al., 2015; Vergara et al., 2016)]. Additionally, there have been no studies to have employed machine learning to classify comorbid PTSD and mTBI. A notable contribution of our work is that we performed machine learning classification, and found that accuracies obtained using network measures were significantly higher (∼10% more) than non-imaging measures. Interestingly, we found that the network measures of the same four aforementioned paths (MFG → insula, insula → amygdala, amygdala → hippocampus, hippocampus → precuneus) resulted in the highest classification accuracy. They were identified to be the top features of diagnostic prediction, in addition to being identified as statistically significant in accordance with our hypothesis, as also being behaviorally relevant through associations with neurocognitive and symptom scores. Each of these attributes were determined in a data-driven fashion from network properties of the entire brain, without imposing any priors or biases. In addition, SVR analysis showed that PCL5 and NSI scores predicted using these network properties could explain 72% and 66% variance in measured symptom severity, respectively. These observations demonstrate that these network-level markers have potential as high-quality biomarkers of the neurobehavioral characteristics of PTSD and PCS. Our network-level features satisfy three out of four conditions posited by Woo and Wager (2015) as necessary to be a good biomarker (diagnosticity, deployability, and interpretability). With regard to the fourth condition (generalizability), based on suggestions by Woo and Wager (2015), we issue an open call for researchers to share clinical data with us for validating our classifier using their data.

Our work presents some notable methodological contributions. While modeling of dynamic connectivity has been prevalent for a while (Hutchison et al., 2013), the modeling of dynamic properties of complex-network measures is in its nascent stages. Graph theoretic measures provide additional characterization of the connectomic brain, which is not available through pairwise connectivity modeling (Rubinov and Sporns, 2010), hence the development and advancement of dynamic complex-network modeling (similar to dynamic connectivity modeling) is important and necessary for brain imaging. A few studies have probed on this topic. Zalesky et al.’s (2014) work was one of the first major studies on dynamics of graph metrics, wherein they introduced the modeling of time-varying graph measures. Liao et al. (2015) later explored the structural substrates of time-varying graph metrics, whereas Chiang et al. (2016) presented a technique for studying temporal stationarity of graph metrics. Chen et al. (2016) took forward these developments to study the dynamics of the salience network’s spatiotemporal organization, while Betzel et al. (2016) studied the correspondence between dynamics of connectivity and dynamics of modularity. These studies have demonstrated the use of time-varying graph metrics in different ways; however, as Preti et al. (2016) have pointed out in their review, prior studies have focused on only two metrics, efficiency and modularity. None of these studies integrates information from both static and time-varying graph metrics, nor have they probed into the dynamics of graph metrics obtained from directional connectivity. Our study is an advancement over these prior works, in that we present a technique to compute time-varying global, nodal as well as connection-level metrics of segregation and integration from effective connectivity networks, additionally presenting a novel framework for integrating the variability in dynamic network information with static network information to study three different cohorts (two psychiatric populations and a control group). In our opinion, this is a notable advancement in the field. Our contribution is broad and robust to accommodate the specific requirements of different varieties of brain imaging studies.

This study integrates several dimensions within a single framework, as follows: (i) Connectivity modeling as well as complex network modeling, (ii) segregation (node-level) as well as integration (connection-level), (iii) static as well as dynamic connectivity, (iv) EC modeling, especially dynamic EC being a recent advancement, (v) PTSD as well as comorbid PCS + PTSD, and (vi) statistical analysis as well as machine-learning based predictive analysis. It is notable that this is the first fMRI study to utilize either effective connectivity or dynamic connectivity or static/dynamic complex-network modeling based on effective connectivity in either PTSD or PCS or the comorbid condition; and one among a few studies to have utilized machine learning in either of these disorders. Additionally, since our findings were based on an overlap/intersection of results with the PTSD and the PCS + PTSD groups, the observations and conclusions are also relevant to the characterization of PTSD alone. We intend to convey that our novel framework is relevant to the study of any cognitive domain, psychiatric or neurologic condition. We urge researchers to employ this framework for enhancing the understanding other disorders and cognitive domains.

Finally, we present several caveats and limitations of our work, demanding careful interpretation of the findings, as also providing suggestions for future works: (1) Participants sustaining an added burden of PCS in addition to PTSD displayed higher symptom severity in comparison to those with PTSD alone. Though there is limited imaging literature on comorbid PTSD and PCS, we speculate that: (i) PTSD-related brain aberrations that were already prevalent before developing PCS would be aggravated by the added burden of a prior mTBI, *or*, (ii) alleviated functional neural aberrations corresponding to elevated symptom severity would be a consequence of the participants sustaining an mTBI with subsequently or concomitantly being exposed to a traumatic experience, in comparison to participants who were exposed to psychological trauma alone. Untangling the underlying cause-effect relationships in comorbid PTSD and PCS could be an aim of future experimental designs, in order to confirm either of the two scenarios. (2) Though we compare and discus the common and distinguishing neural phenotypes of PTSD and mTBI, it must be noted that our study population did not consist of a pure mTBI/PCS group; rather it consisted of a group with elevated PTSD symptoms (pure PTSD group) and a comorbid group diagnosed with both mTBI/PCS and PTSD, using which the common and distinguishing neural phenotypes of PTSD and mTBI/PCS were derived through our novel framework (Figure 1). (3) Military participants with CES were part of our study cohort. This is an invaluable strength of our work since it provides a more representative control group. Recent works have found differences between healthy civilians and healthy combat personnel with resting-state fMRI connectivity (Kennis et al., 2015), “potentially due to military training, deployment, and/or trauma exposure.” Hence, future studies could verify if our findings are equally applicable to civilian or non-combat-related PTSD and PCS. (4) With only male soldiers being considered in this study, our findings are not directly generalizable to female soldiers. (5) During RCE-SVM classification, our entire dataset was split into training (80%) and testing/validation (20%) data sets, resulting in about seventeen participants (20% of 87 participants) in the testing set. This is not a relatively large number for an fMRI connectivity study. (6) Given the heterogeneous patterns in PTSD and mTBI, the number of subjects used in this study is relatively small, which raises concerns about the reproducibility of our results. Our findings must thus be interpreted with certain degree of caution. Additionally, we invite researchers to replicate our study design in larger sample sizes to assess reproducibility of our findings. To determine clinical utility of the findings for diagnosis, the findings must be replicated on a larger sample that is representative of the target population in terms of ethnicity, gender, etc. (7) Given the uncontrolled nature of resting state (Hurlburt et al., 2015), it is not possible to determine whether resting-state connectivity differences between groups are driven by differences in the “type of mind wandering” exhibited by controls versus those with disorder, rather than an inherent “baseline” difference. It is possible that the scanning session had captured the brain while being engaged in the symptomatic state itself rather than, or perhaps in addition to, capturing the underlying physiological weaknesses that putatively caused the symptoms. This issue could be specific to only some clinical populations like ours, where symptoms often manifest during periods of idle thought. It is not possible to completely untangle this problem with the data we have. However, future studies could employ methods such as “descriptive experience sampling” (Hurlburt et al., 2015) in order to characterize the “type of mind wandering” in PTSD versus controls so as to ascertain whether such differences might underlie alterations in resting state connectivity. (8) With our fMRI data being cross-sectional, there is scope for longitudinal studies to develop similar hypotheses over the advancement, recovery and rehabilitation phases of the clinical groups. In addition, it would be an appropriate test for validating the four pivotal network paths underlying information integration (L_MFG → L_Insula, L_Insula → L_Amygdala, L_Amyg → R_Hippocampus, R_Hippocampus → L_Precuneus) as candidate imaging biomarkers for PTSD, and comorbid PCS and PTSD.

## Ethics Statement

This study was conducted in accordance with the Declaration of Helsinki, and the procedures were approved by the Auburn University’s Institutional Review Board (IRB) as well as the Headquarters United States Army Medical Research and Materiel Command, IRB (HQ USAMRDC IRB). Written informed consent was obtained from all the participants. Material has been reviewed by the Walter Reed Army Institute of Research. There is no objection to its presentation and/or publication. The opinions or assertions contained herein are the private views of the author, and are not to be construed as official, or as reflecting true views of the Department of the Army or the Department of Defense. The investigators have adhered to the policies for protection of human subjects as prescribed in AR 70–25.

## Author Contributions

DR, MD, JK, TD, and GD conceived the work and wrote the manuscript. DR, MD, JK, and GD developed the methodology. TD and GD provided the resources and supervised the entire work. MD was the project PI.

## Conflict of Interest Statement

The authors declare that the research was conducted in the absence of any commercial or financial relationships that could be construed as a potential conflict of interest.
