# Supplementary material for: Dynamics of Segregation and Integration in Directional Brain Networks: Illustration in Soldiers With PTSD and Neurotrauma
Source: Front Neurosci. 2019 Aug 23;13:803. doi: 10.3389/fnins.2019.00803 (PMC6716456; doi:10.3389/fnins.2019.00803)
Supplement: Supplementary file 1 [file Data_Sheet_1.pdf]

SUPPLEMENTAL INFORMATION

Dynamics of Segregation and Integration in Directional Brain Networks: Illustration in Soldiers with PTSD and Neurotrauma

D Rangaprakash[1,2], Michael N Dretsch[3,4,5], Jeffrey S Katz[1,5,6,7], Thomas S Denney Jr.[1,5,6,7] and Gopikrishna Deshpande[1,5,6,7,8,9,*]

[1] AU MRI Research Center, Department of Electrical and Computer Engineering, Auburn University, Auburn, AL, USA

[2] Departments of Radiology and Biomedical Engineering, Northwestern University, Chicago, IL, USA

[3] U.S. Army Aeromedical Research Laboratory, Fort Rucker, AL, USA

[4] US Army Medical Research Directorate-West, Walter Reed Army Institute for Research, Joint Base Lewis-McCord, WA, USA

[5] Department of Psychology, Auburn University, Auburn, AL, USA

[6] Alabama Advanced Imaging Consortium, AL, USA

[7] Center for Neuroscience, Auburn University, AL, USA

[8] Center for Health Ecology and Equity Research, Auburn University, Auburn, AL, USA

[9] Department of Psychiatry, National Institute of Mental Health and Neurosciences, Bangalore, India

***Correspondence to:**

Gopikrishna Deshpande, Ph.D.

560 Devall Dr, Suite 266D,

Auburn University, Auburn, AL 36849, USA

Tel.: +1 334 844-7653; Fax: +1 334 844-0214

E-mail: gopi@auburn.edu

SI-1. Introduction: additional points

Granger causality (GC) and dynamic causal modeling (DCM): In our study, we employed GC to quantify effective connectivity. As a data-driven approach, GC has no requirement for the specification of connectivity priors as in DCM [1] [2] [3] [4]. While DCM is relevant in a wide range of applications, for whole-brain connectivity it is practically impossible to build a DCM model with connectivity priors since it would result in computational intractability. These reasons motivated our choice of GC.

Comparing our study with another recent study on complex network modeling in PTSD/mTBI: A recent study reported complex-network characterization in PTSD and mTBI [5]. However, they considered neither directed networks nor dynamic variations in network properties, meaning that our work is looking at a fundamentally different characterization, providing novel insights that cannot be obtained through static or non-directional connectivity. Consequently, their work also did not present a coherent and comprehensive aberrant network structure that could explain PTSD and mTBI symptoms, possibly because they used only global measures of integration that do not give connection-level characterization, and they did not use directional connectivity. Rather they identified certain nodes with reduced static non-directional segregation, which correlated with certain relevant behaviors, whose usefulness and applications are different from that aimed in this work.

SI-2. Supplemental Methods

SI-2.1. Psychological health measures

PTSD Checklist-5 (PCL5, [6]*)*: The PCL5 is a 20-item self-report measure that assesses DSM-V symptoms of PTSD. The PCL5 serves a variety of purposes, including making PTSD diagnoses, monitoring symptom change during and after treatment, and screening individuals for PTSD. Items are rated with a 5-point Likert scale; 1="Not at all" to 5="Extremely". An aggregate score (range: 20-100) is obtained by summing the scores from all the 20 items, with a cut-score of 38 for a precursory diagnosis of PTSD [7].

PCL5 scores were significantly different ($F(2,85)=101.65$, $p=3.64\times10^{-44}$) between control group and the PTSD and PCS+PTSD groups combined. The reason for such a comparison was

that PTSD is the common condition between the PTSD and PCS+PTSD groups, with the PCL5 score measuring only PTSD symptom severity.

Neurobehavioral Symptom Inventory (NSI, [8]*)*: The NSI is a 22-item self-report measure designed to assess post-concussive symptoms in persons sustaining an mTBI. Participants rate the severity of every symptom (within the past month) on a 5-point Likert scale ranging from 0 (none) to 4 (very severe). A total score (range: 0-88) is obtained by summing scores of the 22 items. NSI scores were significantly different ($F(2,85)= 49.79$, $p=1.32\times10^{-29}$) between PCS+PTSD group and the PTSD and control groups combined.

CNS-Vital Signs® (CNS-VS, [9]*):* CNS-VS is a computerized neurocognitive assessment battery. We used five CNS-VS sub-tests (verbal memory, Stroop test, symbol digit coding, shifting attention test and continuous performance test). The following CNS-VS domain scores were then calculated: verbal memory (VM), reaction time (RT), complex attention (CA), cognitive flexibility (CF), processing speed (PS), and executive functioning (EF). Domain scores possess a mean of 100 and standard deviation of 15. They were averaged to form a single aggregate score called neurocognitive composite index (NCI) [9].

SI-2.2. Procedures

Upon arrival at Auburn University's MRI Research Center for the scheduled appointment, participants were re-consented and re-screened for eligibility to ensure full comprehension of the study's procedures, their rights and benefits. Participants finished a computerized neurocognitive battery of questionnaires and tests assessing medical history, demographics and psychological health. Once completed, the participants were escorted to the MRI suite for imaging. After completion, participants were provided a check for up to $250 to compensate for their time commitment.

Two identical but separate resting-state fMRI scans were performed for every participant and processed independently, thus providing 174 sessions of data for the 87 participants, which mathematically boosted the statistical power of our analysis beyond what would have been available from single scans of 87 participants, given that statistics were performed with connectivity values that were double in number (per connectivity path) in comparison with the

total number of participants per group. The two scans were performed on the same day, during the same visit of the soldier to the MRI Research Center. However, they were not done back to back, since a T1 MPRAGE sequence was executed between them.

SI-2.3. Complex-Network Analysis

We first describe the network measures of segregation and integration, and then explain how they were used in the context of this work. As noted earlier, we dealt with weighted directed networks in this work. Weighted, because we did not binarize our connectivity matrices, since binarizing requires choosing an arbitrary threshold value that might bias the results in faulty ways. There are approaches to binarize reliably [10], which essentially involves using a range of arbitrary thresholds and either choosing one of them based on some mathematical criteria or reporting findings for an entire range of thresholds. However, given the complexity of our hypothesis, we chose to use weighted networks instead, which is considered acceptable [10].

Functional segregation was quantified using transitivity (global measure, one value for whole brain per participant), clustering coefficient and local efficiency (both local measures, one value per node/region per participant). Functional integration was quantified using global efficiency (global measure), shortest path length and edge betweenness (both local measures, one value per connection per participant). We obtained source codes for these measures from the Brain Connectivity Toolbox (April 2014 release) [10], and implemented the entire pipeline in the Matlab® platform through custom codes. We explain each of these measures here (for weighted directed networks only), while a more detailed account can be found in [10].

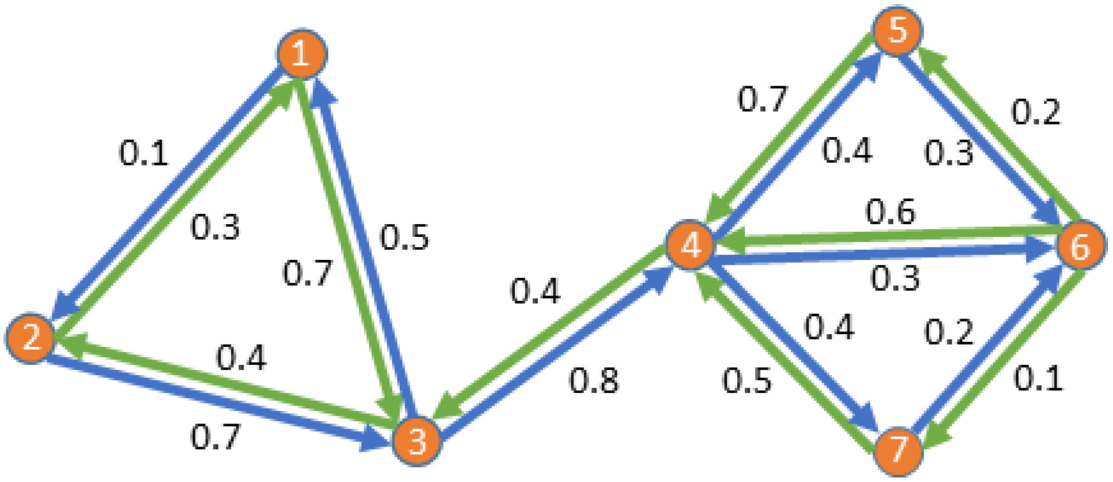

Fig.S1. *A simple example network used to explain several complex-network measures numerically. The node numbers are marked within the node. The connectivity weight for each directional connection are marked next to the arrow. This network contains two sub-networks or modules (nodes 1,2,3 and nodes 4,5,6,7), which are connected through pivotal connections between node-3 and node-4.*

We first define certain basic network entities. All measures are explained numerically using a simplified example (see **Fig.S1**). The degree of a node is the sum of all the connectivity weights associated with a node. Given the set of all nodes (brain regions or ROIs) *N* and connectivity weights (EC value) w_{ij} for path *i-to-j (i,j)*, the degree of a node is defined as,

$$Out\ degree\colon k_i^{out} = \sum_{j\in N} w_{ij};\ \ In\ degree\colon k_i^{in} = \sum_{j\in N} w_{ji} \qquad (1)$$

In the example (**Fig.S1**), the degrees for node-4 are $k_4^{in} = 2.6,\ k_4^{out} = 1.5$, while that for node-2 are $k_2^{in} = 0.5,\ k_2^{out} = 1.0$. This shows that node-4 is a stronger node and is predominantly driven by other nodes while node-2 is a weaker node and predominantly drives others. The weight of a “no connection” is taken as zero. The number of triangles around a node is the basis for measuring segregation, which informs about how well the neighbors of a node are well-connected neighbors themselves, which characterizes how well-connected a sub-network is. The number of triangles around a node is defined as the geometric mean of the triangles around node *i* as,

$$t_i = \frac{1}{2}\sum_{j,h\in N}\left[(w_{ij}+w_{ji})(w_{ih}+w_{hi})(w_{jh}+w_{hj})\right]^{1/3} \tag{2}$$

In the example (subscripts indicate node number), $t_3 = 0.4,\ t_6 = 1.3$. If the connection between nodes 4 and 5 is removed and inserted as the connection between nodes 4 and 1, we get $t_3 = 1.6,\ t_6 = 0.5$, which is a reversal from the former case.

The shortest path length (SPL) is a basis for measuring integration, and is estimated as the smallest sum of inverse path-weights from node i to j. It is a measure of how easy it is to reach node j from node i. The shortest path $g_{i\to j}$ is usually determined using Dijkstra's algorithm [11]. The shortest path length is determined as,

$$d_{ij} = \sum_{w_{uv}\in g_{i\to j}} {}^{1}/_{w_{uv}} \tag{3}$$

In the example (**Fig.S1**), $d_{1\to7} = 5.2,\ d_{7\to1} = 6.5$, indicating that it is shorter (easier) to communicate from node 1 to 7 than from node 7 to 1. The SPL is a supremely important network measure, and is analogous to meta-connectivity, because it represents indirect connections between regions which are directly not connected with pairwise connectivity modeling (or weakly connected). For example, nodes 1 and 7 are not directly connected, but SPL makes it possible to quantify indirect relationships between them through other regions.

Measures of segregation:

Transitivity is a global measure indicating the average percentage of triangles (clusters) associated with the nodes compared to the total strength of all connections. It is a global measure of overall efficiency of local processing in the brain, which is defined as,

$$T = \frac{\sum_{i\in N} t_i}{\sum_{i\in N}\left[\left(k_i^{in}+k_i^{out}\right)\left(k_i^{in}+k_i^{out}-1\right) - 2\sum_{j\in N} w_{ij}w_{ji}\right]} \tag{4}$$

In the example (**Fig.S1**), simple computation yields T=0.22. If nodes 4 to 7 were to be removed, we get T=0.39 since nodes 1-3 form neat triangles. If nodes 1 to 3 were to be removed, we get

T=0.28; lower since nodes 5 and 7 are not connected. The entire network has a smaller transitivity value since the connection from nodes 3 to 4 is not part of any triangle/cluster.

Clustering coefficient (*CC*, a local measure) gives a transitivity-type characterization for every node. It is the ratio of all triangles around a node to the total sum of all connectivity weights associated with the node.

$$CC_i = \frac{t_i}{\left[\left(k_i^{in} + k_i^{out}\right)\left(k_i^{in} + k_i^{out} - 1\right) - 2\sum_{j \in N} w_{ij} w_{ji}\right]} \tag{5}$$

In the example (**Fig.S1**), $CC_3 = 0.13$, $CC_5 = 0.45$, because about one-thirds of node-3's connections do not form a triangle at all, while all of node-5's connections form triangles and with high strengths. Clearly, node-5 is associated with more specialized processing.

Local efficiency (*EffLoc*, a local measure) is closely related to *CC*. Essentially, if a given node has powerful neighbors that are involved in several shortest paths, then the node has high *EffLoc*, indicating that the node is important in the sub-network for specialized processing.

$$EffLoc_i = \frac{\sum_{j,h \in N, j \neq i}\left[(w_{ij} + w_{ji})(w_{ih} + w_{hi})\left([d_{jh}(N_i)]^{-1} + [d_{hj}(N_i)]^{-1}\right)\right]}{\left[\left(k_i^{in} + k_i^{out}\right)\left(k_i^{in} + k_i^{out} - 1\right) - 2\sum_{j \in N} w_{ij} w_{ji}\right]} \tag{6}$$

Where $d_{jh}(N_i)$ is the shortest path length between *j* and *h* which contains only the neighbors of node *i*. In the example (**Fig.S1**), $EffLoc_3 = 0.15$, $EffLoc_5 = 0.45$. While *CC* and *EffLoc* usually give similar (but not same) results, their interpretations are different. In this work, along with transitivity as the global measure, we employed both *CC* and *EffLoc* as local measures, which are the two popularly used local measures of segregation. We took an overlap (intersection) of the final significant group differences for the two measures, so that the affected nodes had differences in both the measures, thus providing more conservative results with a broader interpretation.

Measures of integration:

Global efficiency (*EffGlob*) is a global measure indicating the aggregate ease of communication in the entire network. It is defined as the average inverse shortest path length of

the complete network. That means, if the shortest paths in the network are shorter (easier to communicate) on average then we get a larger global efficiency.

$$EffGlob = \frac{1}{n}\sum_{i\in N} \frac{\sum_{j\in N, j\neq i}\left(d_{ij}\right)^{-1}}{n-1} \tag{7}$$

Where *n* is the total number of nodes in the network. In the example shown in **Fig.S1**, *EffGlob*=0.32. Now if we were to remove the connections between nodes 1 and 2, we would get *EffGlob*=0.32 (unchanged), because it is a localized connection that does not have much role in information integration. However, if we were to remove the connections between nodes 3 and 4, which is the key link between the two sub-networks, we would get *EffGlob*=0.18.

Edge betweenness (*EB*) is a local measure obtained for each connection. For a given connection, it measures the number of all shortest paths in the entire network that contain the given connection. That means, if the connection were an important link in the network, then a large portion of shortest paths would go through it, giving a high value of *EB*. It is evaluated through a variant of Dijkshra's algorithm [10]. In our example (**Fig.S1**), $EB_{1\to 2} = 0,\ EB_{4\to 6} = 4,\ EB_{6\to 4} = 6,\ EB_{3\to 4} = 12$. These values are intuitive since path 1→2 has little role in integrating the rest of the network, while the paths between nodes 4 and 6 have a role restricted within the sub-network (with 6→4 being stronger than 4→6). The path between nodes 3 and 4, however, is pivotal to the efficient communication between the sub-networks and integration of information, which is why we observe the highest *EB* for this path.

SI-2.4. Machine learning classification analysis

Here, we explain the RCE-SVM classification technique [12] in detail. The data was first split into training data (patterns in the data are learned from this) and testing/validation data (the learned pattern is independently tested with this to assess the quality of learning). Significant group differences were then identified for all three comparisons (PTSD vs PCS+PTSD, control vs PCS+PTSD, and control vs PTSD) from the training data only, using a threshold of $p<0.05$ (controlled for age, education, race and head motion). An uncorrected significance threshold was used ($p<0.05$) since we wanted to be liberal about which features were fed into the classifier, thus

having the classifier itself choose the most predictive features. Next, we identified overlapping network measures between the three comparisons, across all network measures, which formed the input features to the classifier. This initial filtering is found to enhance the quality of classification [13] by ensuring that non-discriminatory features are not input to the classifier.

Our choice of support vector machine (SVM) [14] for classification was driven by its wide applicability and acceptance in several fields including neuroimaging [15]. Earlier studies have illustrated that the use of discriminatory features enhances SVM classification performance [12] [13]. Thus, we employed the wrapper method of recursive cluster elimination (RCE), which iteratively eliminates features to minimize prediction error, wherein feature selection and feature classification steps are embedded together. The RCE-SVM classification technique's main steps are the clustering step, the SVM-scoring step and the RCE step. Features initially fed into the classifier were divided into training and testing datasets. The classifier was trained on the training data, while the testing data was entirely kept blinded to the classifier. Once training was completed, the testing data was fed to the classifier to obtain the classification accuracy, which ensured generalizability of results.

In clustering step, the k-means algorithm was utilized to cluster training data into 'N' clusters. Initially the number of clusters was set as the number of features, and was then iteratively reduced by one, until no empty clusters remained. The value of 'N' obtained through this served as the initial 'N' for the RCE-SVM loop. In the SVM-scoring step, linear SVM was employed and each cluster was scored based on its ability to differentiate between groups. To assess cluster performance, training data was randomly partitioned into six non-overlapping subsets of equal sizes (i.e. six folds). Using five of those subsets, SVM was trained and performance (accuracy) was computed using the remaining subset. All possible partitions were generated by repeating the clustering cum cross-validation procedure a hundred times. For each of the hundred repetitions, classification accuracy was obtained using the testing data.

Using the outcome of these hundred repetitions and six folds for each repetition, the cluster's score was obtained as the average accuracy. Bottom 20% of low-scoring clusters were rejected in the RCE step. Remaining features were then merged and the value of 'N' was reduced by 20%. This ascertained that only a few top-classifying features qualified for the next iteration. The clustering step, SVM-scoring step and the RCE-step were iteratively repeated again. After each

such iteration, classifier performance was computed using reduced number of features compared to the earlier iterations. Once the number of clusters reached two, the process was terminated. **Fig.S2** illustrates the RCE-SVM procedure with a flowchart. The complete separation of training and testing data sets eliminates bias in the calculation of classification accuracy [16]. Further, the few features obtained in the final two clusters would then be those with highest discriminative ability, hence carrying predictive value for diagnosis. Complete information on the RCE-SVM algorithm could be obtained from previous reports [12] [17].

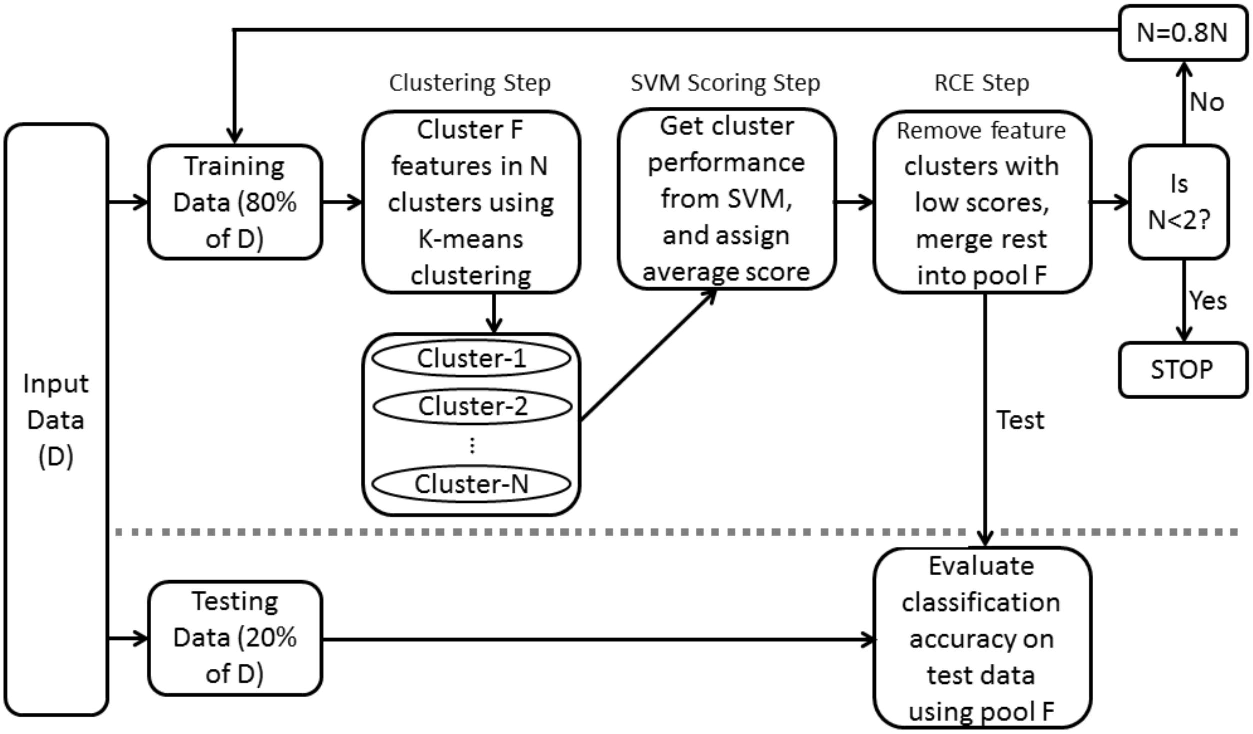

Fig.S2. *Flowchart describing the recursive cluster elimination based support vector machine (RCE-SVM) classification procedure*

SI-3. Supplemental Results

SI-3.1. Observations using a different brain parcellation instead of Craddock-200

We repeated our analysis pipeline by using the Shen functional brain parcellation [18] instead of the Craddock-200 atlas (identical statistical methods and thresholds were used in the repeated

analysis). We found very similar results using the Shen atlas as compared to the Craddock-200 atlas. Specifically, we found altered functional segregation within ROIs in the middle frontal gyrus (MFG), medial prefrontal, anterior insula, hippocampus and visual regions. We found altered functional integration from left MFG to the left insula, left amygdala and left hippocampus, to the lateral parietal cortex – these were altered between all three groups. We found altered integration from the same prefrontal regions to visual and lateral parietal, which were significantly different for control vs PTSD and control vs PCS+PTSD comparisons, but not for PTSD vs PCS+PTSD comparison. These results are essentially the same as those observed using the Craddock-200 atlas. Machine learning results also showed the same trend of higher accuracy using graph measures as compared to non-imaging measures; a classification accuracy of 81.16% was obtained (which is close to the 81.37% accuracy obtained using Craddock-200 atlas). The classification accuracies obtained with the two atlases were not significantly different (p=0.56). Since we obtained similar results with two different atlases, it is reassuring that our results are not highly specific to the Craddock-200 parcellation.

SI-3.2. Observations using eigenvariate time series data instead of mean time series

In this study, mean time series was obtained (post voxel-level deconvolution) from 3D+time fMRI data using the Craddock-200 atlas, as described in the main manuscript. The practice of computing mean time series from ROIs has been followed in fMRI studies since a long time (for example, [19] [20] [21] [22] [23]). Each ROI may have a few hundred/thousand voxels; hence, given that the purpose of ROI time series extraction is to obtain one time series from hundreds/thousands of voxel time series, it is an obvious step to take the average of all voxel time series to obtain the ROI time series. This explains its widespread use. We have used mean time series in several of our recent publications as well [24] [25] [26] [27]. That being said, we also acknowledge that some studies have questioned the approach of averaging the time series [28]. Hence, as an alternate approach, we obtained eigenvariate time series [29] instead of mean time series and repeated the analysis. Across all subjects and voxels, the correlation between mean time series and eigenvariate time series was 0.948±0.044 (significantly correlated in 100% of the data, $p<0.05$), implying that the two approaches resulted in nearly identical time series. We also repeated

the analysis pipeline with eigenvariate time series instead of mean time series and arrived at the same results (same segregation nodes and integration connections were identified).

SI-3.3. Observations using ROI-level deconvolved data instead of voxel-level deconvolved data

In our study, we performed deconvolution on voxel-level time series, and then extracted mean deconvolved time series from that using the Craddock-200 parcellation. An alternate option would have been to extract mean ROI time series first and then perform deconvolution on ROI data. Here we provide justification for choosing the former.

Our choice is driven by the fact that intra-subject spatial variability of the hemodynamic response function (HRF) has been demonstrated before [30] [31]. HRF varies across the brain within an individual, and this variability depends on several factors that modulate neurovascular coupling including blood vessels and neuromodulators within the voxel [32] [33] [34]. These factors could vary sharply across nearby voxels, for example, if an artery is passing through a voxel then that voxel is expected to have a markedly different HRF compared to its neighbors that don't have the artery. This being the case, it is preferable to model HRF variability at the voxel-level to minimize the confound of HRF variability as much as possible, which explains our choice.

Nevertheless, we repeated the analysis pipeline by performing deconvolution on mean ROI time series instead of voxel-level time series. Across all subjects and ROIs, we found that the correlation between voxel-level deconvolved and ROI-level deconvolved ROI time series data was 0.922 ± 0.063 (the correlations were significant in every ROI in every subject). Hence, it appears that, on average, the two approaches result in very similar deconvolved time series. We repeated our analysis pipeline using ROI-deconvolved data (identical statistical analysis and thresholds) and found the same results (same segregation regions and integration connections emerged).

SI-3.4. Supplemental machine learning classification results

In the main text, we presented worst-case classification accuracy results (Fig.9b and Table 3). Here we present **Fig.S3**, showing average classification accuracies instead.

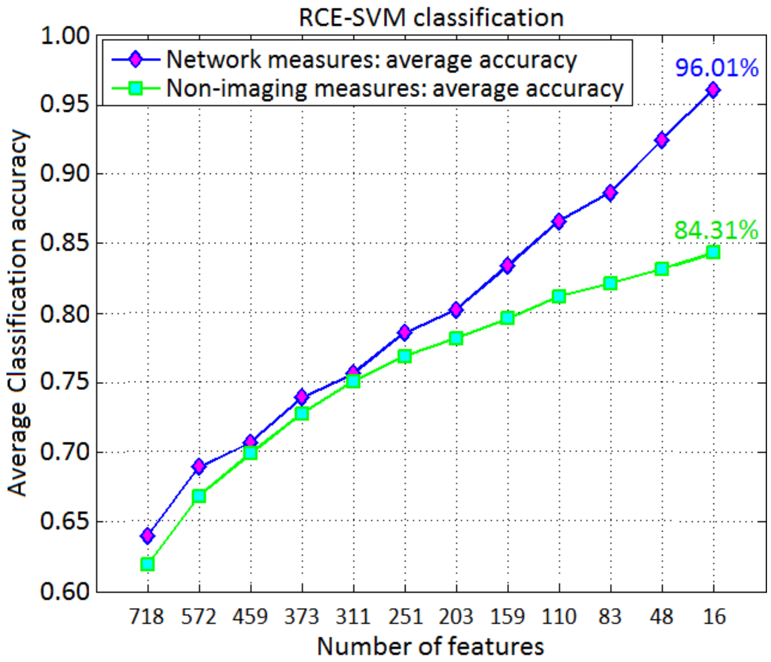

Fig.S3. *Machine learning classification was performed using recursive cluster elimination based support vector machine (RCE-SVM) classifier, to classify between control, PTSD and PCS+PTSD groups. Figure shows average classification accuracies obtained using recursively reducing number of discriminative features (poorer features are successively eliminated). Classification was independently performed with both whole-brain network measures and non-imaging measures. We observed that network measures consistently outperformed NIMs, with about 11% better performance in the final RCE step using top-predictive connectivity features. The trend is highly similar to what was observed with worst-case accuracies.*

REFERENCES

[1] G. Deshpande and X. Hu, "Investigating effective brain connectivity from fMRI data: past findings and current issues with reference to Granger causality analysis," *Brain Connectivity,* vol. 2, no. 5, pp. 235-45, 2012.

[2] A. Roebroeck, E. Formisano and R. Goebel, "Mapping directed influence over the brain using Granger causality and fMRI," *Neuroimage,* vol. 25, no. 1, pp. 230-242, 2005.

[3] G. Deshpande, K. Sathian, X. Hu and J. A. Buckhalt, "A rigorous approach for testing the constructionist hypotheses of brain function," *The Behavioral and Brain Sciences,* vol. 35, no. 3, pp. 148-9, 2012.

[4] K. J. Friston, L. Harrison and W. Penny, "Dynamic causal modelling," *Neuroimage,* vol. 19, no. 4, pp. 1273-302, 20013.

[5] J. M. Spielberg, R. E. McGlinchey, W. P. Milberg and D. H. Salat, "Brain network disturbance related to posttraumatic stress and traumatic brain injury in veterans," *Biological Psychiatry,* vol. 78, no. 3, pp. 210-6, 2015.

[6] B. D. Dickstein, F. W. Weathers, A. C. Angkaw, C. M. Nievergelt, K. Yurgil, W. P. Nash, D. G. Baker, B. T. Litz and the Marine Resiliency Study Team, "Diagnostic Utility of the Posttraumatic Stress Disorder (PTSD) Checklist for Identifying Full and Partial PTSD in Active-Duty Military," *Assessment,* p. pii: 1073191114548683, 2014.

[7] F. W. Weathers, B. T. Litz, T. M. Keane, P. A. Palmieri, B. P. Marx and P. P. Schnurr, "The PTSD Checklist for DSM-5 (PCL-5)," 01 05 2015. [Online]. Available: Scale available from the National Center for PTSD at www.ptsd.va.gov. [Accessed 01 05 2015].

[8] K. D. Cicerone and K. Kalmar, "Persistent postconcussion syndrome: The structure of subjective complaints after mild traumatic brain injury," *The Journal of head trauma rehabilitation,* vol. 10, no. 3, pp. 1-17, 1995.

[9] C. T. Gualtieri and L. G. Johnson, "Reliability and validity of a computerized neurocognitive test battery, CNS Vital Signs," *Archives of Clinical Neuropsychology,* vol. 21, no. 7, pp. 623-43, 2006.

[10] M. Rubinov and O. Sporns, "Complex network measures of brain connectivity: uses and interpretations," *Neuroimage,* vol. 52, no. 3, pp. 1059-69, 2010.

[11] E. Dijkstra, "A note on two problems in connexion with graphs," *Numerische Mathematik,* vol. 1, no. 1, pp. 269-271, 1959.

[12] G. Deshpande, Z. Li, P. Santhanam, C. L. M. Coles, S. Hamann and X. Hu, "Recursive cluster elimination based support vector machine for disease state prediction using resting state functional and effective brain connectivity," *PLoS One,* vol. 5, no. 12, p. e14277, 2010.

[13] R. Craddock, P. Holtzheimer III, X. Hu and H. Mayberg, "Disease State Prediction From Resting State Functional Connectivity," *Magnetic Resonance in Medicine,* p. in press, 2009.

[14] V. Vapnik, The nature of statistical learning theory, New York: Springer, 1995.

[15] L. Wang, Support Vector Machines: Theory and Applications, New York: Springer, 2005.

[16] N. Kriegeskorte, W. Simmons, P. Bellgowan and C. Baker, "Circular analysis in systems neuroscience: the dangers of double dipping," *Nature Neuroscience,* vol. 12, no. 5, p. 535–540, 2009.

[17] M. Yousef, S. Jung, L. Showe and M. Showe, "Recursive Cluster Elimination (RCE) for classification and feature selection from gene expression data," *BMC Bioinformatics,* vol. 8, p. 144, 2007.

[18] X. Shen, F. Tokoglu, X. Papademetris and R. Constable, "Groupwise whole-brain parcellation from resting-state fMRI data for network node identification," *Neuroimage,* vol. 82, pp. 403-15, 2013.

[19] K. Vinehout, B. Schmit and S. Schindler-Ivens, "Lower Limb Task-Based Functional Connectivity Is Altered in Stroke," *Brain Connect.,* vol. 9, no. 4, pp. 365-377, 2019.

[20] P. Mulders, P. van Eijndhoven, J. Pluijmen, A. Schene, I. Tendolkar and C. Beckmann, "Default mode network coherence in treatment-resistant major depressive disorder during electroconvulsive therapy," *J Affect Disord.*, vol. 205, pp. 130-137, 2016.

[21] A. Kucyi, M. Hove, J. Biederman, K. Van Dijk and E. Valera, "Disrupted functional connectivity of cerebellar default network areas in attention-deficit/hyperactivity disorder," *Hum Brain Mapp.,* vol. 36, no. 9, pp. 3373-86, 2015.

[22] E. Kehoe, D. Farrell, C. Metzler-Baddeley, B. Lawlor, R. Kenny, D. Lyons, J. McNulty, P. Mullins, D. Coyle and A. Bokde, "Fornix White Matter is Correlated with Resting-State Functional Connectivity of the Thalamus and Hippocampus in Healthy Aging but Not in Mild Cognitive Impairment - A Preliminary Study," *Front Aging Neurosci.,* vol. 7, p. 10, 2015.

[23] S. Bhattacharyya, I. Falkenberg, R. Martin-Santos, Z. Atakan, J. Crippa, V. Giampietro, M. Brammer and P. McGuire, "Cannabinoid modulation of functional connectivity within

regions processing attentional salience," *Neuropsychopharmacology,* vol. 40, no. 6, pp. 1343-52, 2015.

[24] G. Deshpande, L. Libero, K. Sreenivasan, H. Deshpande and R. Kana, "Identification of neural connectivity signatures of autism using machine learning," *Frontiers in Human Neuroscience,* vol. 17, p. 7:670, 2013.

[25] D. Rangaprakash, G. Deshpande, T. Daniel, A. Goodman, J. Robinson, N. Salibi, J. Katz, T. Denney and M. Dretsch, "Compromised Hippocampus-Striatum Pathway as a Potential Imaging Biomarker of Mild Traumatic Brain Injury and Posttraumatic Stress Disorder," *Human Brain Mapping,* vol. 38, no. 6, pp. 2843-2864, 2017.

[26] D. Rangaprakash, G.-R. Wu, D. Marinazzo, X. Hu and G. Deshpande, "Hemodynamic response function (HRF) variability confounds resting-state fMRI functional connectivity," *Magnetic Resonance in Medicine,* vol. 80, no. 4, pp. 1697-1713, 2018.

[27] D. Rangaprakash, M. N. Dretsch, W. Yan, J. S. Katz, T. S. Denney and G. Deshpande, "Hemodynamic variability in soldiers with trauma: Implications for functional MRI connectivity studies," *NeuroImage: Clinical,* p. in press, 2017.

[28] C. Langen, R. Muetzel, L. Blanken, A. van der Lugt, H. Tiemeier, F. Verhulst, W. Niessen and T. White, "Differential patterns of age-related cortical and subcortical functional connectivity in 6-to-10 year old children: A connectome-wide association study," *Brain Behav.,* vol. 8, no. 8, p. e01031, 2018.

[29] D. Rangaprakash, C. Bohon, K. Lawrence, T. Moody, F. Morfini, S. Khalsa, M. Strober and J. Feusner, "Aberrant Dynamic Connectivity for Fear Processing in Anorexia Nervosa and Body Dysmorphic Disorder," *Front Psychiatry.,* vol. 9, p. 273, 2018.

[30] D. A. Handwerker, J. M. Ollinger and M. D'Esposito, "Variation of BOLD hemodynamic responses across subjects and brain regions and their effects on statistical analyses," *Neuroimage,* vol. 21, no. 4, pp. 1639-51, 2004.

[31] G. K. Aguirre, E. Zarahn and M. D'esposito, "The variability of human, BOLD hemodynamic responses," *Neuroimage,* vol. 8, no. 4, pp. 360-9, 1998.

[32] G. Buzsáki, K. Kaila and M. Raichle, "Inhibition and brain work," *Neuron,* vol. 56, no. 5, pp. 771-83, 2007.

[33] G. G. Brown, L. T. Eyler Zorrilla, B. Georgy, S. S. Kindermann, E. C. Wong and R. B. Buxton, "BOLD and perfusion response to finger-thumb apposition after acetazolamide administration: differential relationship to global perfusion," *Journal of Cerebral Blood Flow and Metabolism,* vol. 23, no. 7, pp. 829-37, 2003.

[34] J. Duarte, J. Pereira, B. Quendera, M. Raimundo, C. Moreno, L. Gomes, F. Carrilho and M. Castelo-Branco, "Early disrupted neurovascular coupling and changed event level hemodynamic response function in type 2 diabetes: an fMRI study," *Journal of Cerebral Blood Flow and Metabolism*, vol. 35, no. 10, pp. 1671-1680, 2015.
